# Deterministic processes dominate archaeal community assembly from the Pearl River to the northern South China Sea

**DOI:** 10.3389/fmicb.2023.1185436

**Published:** 2023-06-22

**Authors:** Xizheng Lin, Chuanlun Zhang, Wei Xie

**Affiliations:** ^1^School of Marine Sciences, Sun Yat-sen University, Zhuhai, China; ^2^Southern Marine Science and Engineering Guangdong Laboratory (Zhuhai), Zhuhai, China; ^3^Guangdong Provincial Key Laboratory of Marine Resources and Coastal Engineering, Zhuhai, China; ^4^Southern Marine Science and Engineering Guangdong Laboratory (Guangzhou), Guangzhou, China; ^5^Shenzhen Key Laboratory of Marine Archaea Geo-Omics, Department of Ocean Science & Engineering, Southern University of Science and Technology, Shenzhen, China; ^6^Shanghai Sheshan National Geophysical Observatory, Shanghai Earthquake Agency, Shanghai, China

**Keywords:** archaeal community, neutral community model, null model, deterministic processes, stochastic processes, co-occurrence network

## Abstract

Archaea play a significant role in the biogeochemical cycling of nutrients in estuaries. However, comprehensive researches about their assembly processes remain notably insufficient. In this study, we systematically examined archaeal community dynamics distinguished between low-salinity and high-salinity groups in water and surface sediments over a 600-kilometer range from the upper Pearl River (PR) to the northern South China Sea (NSCS). Neutral community model analysis together with null model analysis showed that their C-score values were greater than 2, suggesting that deterministic processes could dominate the assembly of those planktonic or benthic archaeal communities at both the low-salinity and high-salinity sites. And deterministic processes contributed more in the low-salinity than high-salinity environments from the PR to the NSCS. Furthermore, through the co-occurrence network analysis, we found that the archaeal communities in the low-salinity groups possessed closer interactions and higher proportions of negative interactions than those in the high-salinity groups, which might be due to the larger environmental heterogeneities reflected by the nutrient concentrations of those low-salinity samples. Collectively, our work systematically investigated the composition and co-occurrence networks of archaeal communities in water as well as sediments from the PR to the NSCS, yielding new insights into the estuary’s archaeal community assembly mechanisms.

## 1. Introduction

Estuaries account for just 0.4 percent of the worldwide ocean area, but are among the most productive ecosystems (Longhurst et al., 1995; Cloern et al., 2014). They are also a buffer zone for transporting terrestrial silicon, phosphorus, and nitrogen into the ocean, allowing coastal zones to have high primary production (Harrison et al., 2008). Continental freshwater runoff and coastal seawater mix in estuaries, creating physicochemical gradients (Vieira et al., 2007; Bernhard and Bollmann, 2010). These physicochemical gradients, such as a decrease in the organic compounds and nitrogen nutrients levels or an increase in salinity, sulfate, and chloride levels, can have profound effects on microbial community structure (Ait Alla et al., 2006; Webster et al., 2015). Additionally, the continuous accumulation of terrigenous nutrients and microorganisms transported to the estuary increases biodiversity and microbial activity, affecting the biogeochemical and ecological processes of the estuarine ecosystem (Baird et al., 2004; Canfield and Thamdrup, 2009; Webster et al., 2015; Zhou et al., 2017; Liu et al., 2018). The industrialization and urbanization along the river caused severe pollution, eutrophication, and hypoxia in the estuary due to inflows from many different continents.

The Pearl River (PR) is in southern China. Approximately 8.5 × 10^7^ tons of sediment are discharged annually into the northern South China Sea (NSCS), accounting for more than 80% of the total suspended particulate matter into the Pearl River estuary (PRE) (Zhou et al., 2004). Due to the industrialization and urbanization development of the Pearl River Delta, large quantities of terrestrial waste have been dumped into the estuary, causing severe pollution to aquatic and benthic habitats (Huang et al., 2003). Even though microbes play an essential role in the degradation of pollutants, research on the ecological functions of aquatic and benthic microbes in the eutrophic PRE is limited.

In estuaries, archaea make up a large proportion of the microbial community. They have high diversity, and have profound influence on the biogeochemical cycling of estuarine ecosystems. Currently, it has been found that archaea have a variety of functions, such as ammonia oxidation (Könneke et al., 2005), methane metabolism (Orphan et al., 2002), organic matter degradation (Lloyd et al., 2013), and sulfate reduction (Zhou et al., 2019). Recent studies have found that the community structure of estuarine archaea is influenced by salinity, dissolved oxygen, and nutrient levels (Xie et al., 2014; Webster et al., 2015; Liu et al., 2018; Zou et al., 2020a). Along a salinity gradient, the structure of archaeal communities and their potential ecological functions in surface sediments in the PRE have been explored before (Xie et al., 2014; Zou et al., 2020b). Xie et al. (2018) investigated the localized high abundance and potential niche adaptation of Marine Group II archaea in the PRE. Ma et al. (2021) explored the distribution and activity of AOA in the PRE. Previous studies have shown that AOA are dominant archaeal taxa in both estuarine water and sediments (Zou et al., 2020a), however, the distribution of specific subgroups in the estuarine region lacks detailed description. Most previous reports on estuarine archaea have only focused on archaeal community composition and their distribution patterns. Currently, studies on the assembly of archaeal communities are far from enough, especially in estuarine areas.

In aquatic microbial ecology, microbial community assembly, the possible mechanisms that regulate microbial community diversity, is still a poorly understood topic despite its importance (Hanson et al., 2012; Zhou and Ning, 2017; Logares et al., 2018; Chen et al., 2019; Mo et al., 2021). There are two significant and complementary mechanisms for describing and comprehending microbial community assembly, namely niche-based theory and neutral-based theory (Sloan et al., 2006; Bahram et al., 2016). Specifically, niche-based theory argues that microbial communities are formed as a result of both deterministic biotic factors (species interactions, e.g., predation and competition) caused by varying habitat partiality and the fitness of microorganisms, as well as abiotic factors, i.e., environmental factors including salinity, pH, nutrients, etc. (Lima-Mendez et al., 2015; Liu et al., 2015). The neutral theory, on the contrary, claims that it is stochastic processes including birth and death, migration, speciation, and dispersal limitation that determine microbial community structures (Bahram et al., 2016; Chen et al., 2017; Zhou and Ning, 2017), in which a stochastic balance is observed between losses and gains of taxa (Sloan et al., 2006; Östman et al., 2010).

Previous studies have found that deterministic processes played a relatively more important role in entire prokaryotic and archaeal community assembly in mangroves than stochastic processes (Zhang et al., 2019, 2021). Gao et al. (2021) reported that stochastic processes dominated the assembly of the archaeal communities on coastal wetlands, and that stochastic processes became more pronounced with increasing frequency of inundation. Chen and Wen (2021) reported that while subtropical mangrove sediments were invaded by *Spartina alterniflora*, stochastic processes dominated the overall archaeal and bacterial community assembly, with the stochastic effect of bacteria stronger than that of archaea. Estuaries, as land-sea connection points, have unique characteristics different from terrestrial and oceanic environments (McLusky and Elliott, 2004), providing a unique environment for testing the theories of community assembly. Although several research have explored the estuarine archaeal community composition and their influencing factors, few attempts have been made to analyze the relative influence of stochastic verse deterministic processes in estuarine areas.

In this study, we aimed to (1) systematically examine the heterogeneous distribution of total archaea and AOA from water and sediment over a 600-kilometer range from the PR to the NSCS; (2) explore the geographic patterns of archaeal communities; (3) explore co-occurrence relationships and keystone taxa in archaeal communities based on network topological properties; and (4) investigate archaeal community assembly in estuarine environments.

## 2. Materials and methods

### 2.1. Sampling and measurements of physicochemical parameters

**Figure 1:**
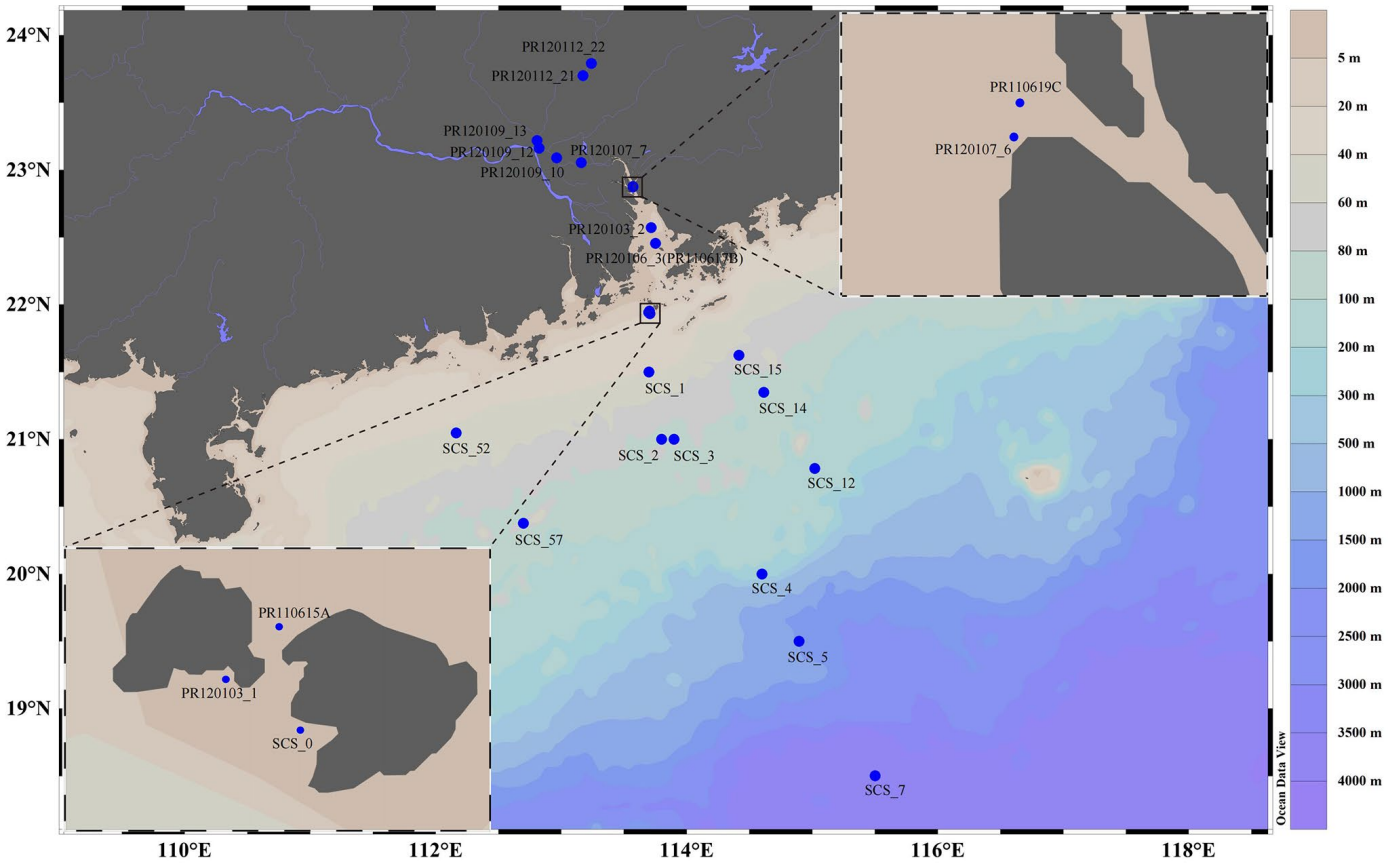
Sampling stations from the Pearl River to the northern south China Sea. Inset, enlarged views of part of the studied areas; blue dots, sampling stations.

Water and surface sediment samples from the PR to the NSCS region (18.501^°^ to 23.791^°^N, and 112.164^°^ to 115.501^°^ E) were collected along a salinity gradient during three cruises in June 2011, January 2012, and October 2012 (Figure 1; Supplementary Table S1). Supplementary Table S1 provides additional information on eight sediment sample data from one of our previous studies (Xie et al., 2014). The uncontaminated seawater was taken from different depths and was sequentially filtered by glass-fiber filters (0.7 μm) to obtain microorganisms, stored on board in liquid nitrogen, and transferred to a laboratory freezer at −80°C for further analysis. Water samples for the determination of inorganic nutrients were collected, filtered through a 0.45 μm cellulose acetate membrane and stored at −20°C until analysis. Immediately after waterbed sampling, a centrifuge tube of 50 mL was used to seal the sediments. Then these tubes were stored on board in liquid nitrogen and transferred to a laboratory freezer at −80°C for further analysis. Before sediment collection, *in situ* measurements of salinity, temperature, and water depth were conducted with a YSI instrument (model YSI 650 MDS, YSI Corporation, United States). Before pore water extraction, samples were stored under −20°C until centrifugating at 4,000 rpm for further analysis. The pore water treatment method was as described in our previous reports (Xie et al., 2014).

### 2.2. DNA extraction, sequencing, and sequence data processing

DNA was extracted using the FastDNA SPIN Kit for Soil (MP Biomedicals, OH, USA) according to the manufacturer’s instructions and preserved at −80°C until further processing. Twenty-eight water samples and 17 sediment samples were selected for pyrosequencing targeting archaeal 16S rRNA (8 sediment data have been published in our previous article (Xie et al., 2014); detailed in Supplementary Table S1), and 20 water samples and 11 sediment samples were selected for pyrosequencing targeting the archaeal *amoA* gene. The Arch_344F/Arch_915R (Gantner et al., 2011), and Arch_amoAF/Arch_amoAR (Francis et al., 2005) primers were used for PCR of the archaeal 16S rRNA and AOA-*amoA*, respectively. Pyrosequencing was performed on the Roche GS FLX+ (454) system, raw data was extracted from 454 data formats by Mothur pipeline (version 1.29.2) (Schloss et al., 2009) and then analyzed using the QIIME2 standard pipeline (version 2020.6) (Bolyen et al., 2019). Specifically, the first step was to filter raw data to remove reads with low quality and ambiguity. The remaining high-quality sequences of 16S rRNA and *amoA* were clustered into operational taxonomic units (OTUs) with the command “qiime vsearch cluster-features-denovo” with 97 and 96% sequence identity thresholds, respectively. Taxonomic assignment for archaeal 16S rRNA representative sequences was obtained by the SILVA database (release 138; Quast et al., 2013). Phylogenetic clades of AOA were affiliated following past studies (Schleper et al., 2005; Alves et al., 2018).

### 2.3. Statistical analysis

Alpha and Beta diversity were calculated from the 16S rRNA and *amoA* datasets using uniform subsampled depths for 1732 and 1,283 sequences, respectively. Rarefaction curves and alpha diversity index for each sample were calculated using the diversity function within R package “vegan” (version 3.6.1; Oksanen et al., 2020). Sample clustering was employed at the archaeal OTU level and the unweighted pair-group method with arithmetic means (UPGMA) based on the Bray–Curtis similarity algorithm. Analysis of similarities (ANOSIM) was implemented using PAleontological Statistics (PAST, version 3.16) to test the differences among groups. Principal component analysis (PCA) was conducted to identify the dissimilar relationships among samples in this study based on the total archaeal community, and AOA subcommunity composition at the OTU level in the STAMP program (Parks et al., 2014). To determine the phylogenetic clades of *amoA* sequences, we obtained reference sequences and tree from a past study (Alves et al., 2018), performed the multiple sequences alignment with MAFFT v7 (Katoh and Standley, 2013), the phylogenetic placement of *amoA* sequences with EPA-ng (Barbera et al., 2019), and visualization with ITOL v5 (Letunic and Bork, 2021). Environmental heterogeneity was estimated by computing the average dissimilarity between sites (Huber et al., 2020) based on abiotic variables by using a Euclidean distance.

### 2.4. Neutral community model

The neutral community model was used to evaluate the effects of stochastic processes on archaeal community assembly (Sloan et al., 2006), and nonlinear least-squares methods were used to determine the best fit between OTU occurrence frequencies and their relative abundance (Elzhov et al., 2016). Östman’s method was used to calculate the *R*^2^ value, which indicates the goodness of fit of the model to datasets (Östman et al., 2010). As *R*^2^ approaches 1, the community composition follows a completely stochastic process. *R*^2^ can be ≤0 when the model does not match the community composition.

### 2.5. Null model

To weigh the relative importance between deterministic verse stochastic processes to the archaeal communities, we evaluated the deviation of each observed metric from the average of the null model (checkerboard score (C-score)) to test the overdispersion or underdispersion of archaeal communities (Stone and Roberts, 1990). The results were normalized so that they could be compared among groups using the standardized effect size (SES). SES was computed under the null model after converting the sequence table to a binary matrix with presence (1) and absence (0) (Gotelli and McCabe, 2002). The SES for the C-score was calculated by dividing the difference between the observed and the mean of the stimulated index by the standard deviation of the stimulated index (Crump et al., 2009). Overdispersion or underdispersion is defined as an SES value that is greater or lower than the expected null value. The strength of the influence of deterministic processes on communities is understood as the magnitude of SES (Swenson, 2014). The dominance of stochastic processes is indicated by SES values between −2 and 2, whereas SES values less than −2 or more than 2 show that deterministic processes (i.e., aggregation and segregation) are more important in community assembly than stochastic processes (Stone and Roberts, 1990; Mo et al., 2021). We calculated the C-score based on the sequential swap randomization algorithm and 30,000 simulations with a R-package named “EcoSimR” (R Core Team, 2013). In addition, to assess how stochastic and deterministic processes affect archaeal community assembly, build-in function “niche.width” of R package “spaa” was used to calculate Levins’ niche breadth (B) index as previous studies (Oksanen et al., 2013). OTUs with higher B values indicated a wider range of habitat niche breadth. Community-level B value (Bcom) was obtained by averaging the B values of all taxa occurring in this community (Wu et al., 2018; Jiao et al., 2020). At the community level, archaeal communities with wider niche breadths should exhibit greater metabolic flexibility than those with narrower niche breadths (Pandit et al., 2009; Wu et al., 2018; Jiao et al., 2020).

### 2.6. Network construction

The co-occurrence networks were constructed using the Molecular Ecological Network Analysis Pipeline (MENA). Only OTUs that were found in more than two samples and with a proportion above 0.2% were retained to reduce complexity. Spearman’s correlation coefficient between OTUs was calculated, and relationships were classified as valid when the correlation coefficient |r| was greater than 0.6 with a *p* value less than 0.05. The network was visualized in Gephi version 0.9.2 (Bastian et al., 2009). Moreover, 1,000 Erdös-Réyni random networks, with the same number of nodes and edges as the real network, were calculated using the R package “igraph,” where each edge is assigned the same probability to each node (Erdos and Rényi, 1960). To further describe the topological parameters, the clustering coefficient, average path length, and modularity of both real and random networks were calculated and compared. Finally, we removed nodes in the static network with a random repetitive process to observe the changes in the natural connectivity of nodes to assess network robustness (Peng and Wu, 2016).

## 3. Results

### 3.1. Archaeal community composition and diversity

As indicated by the rarefaction curves for total archaeal communities and AOA subcommunities, all samples reached a plateau at the sequencing depth applied (Supplementary Figure S1). The number of reads were sufficient for a robust statistical analysis. According to the clustering results, all water samples were divided into two categories: the low-salinity-water group (salinity <14.58 ‰, nitrate ranged from 67.47 to 494.4 μM, silicate ranged from 1.13 to 157.64 μM, and phosphate ranged from 4.155 to 26.885 μM) and the high-salinity-water group (salinity >21.08 ‰, nitrate ranged from 0.139 to 149.223 μM, silicate ranged from 0.22 to 65.031 μM, and phosphate ranged from 0.016 to 6.481 μM; Supplementary Figure S2A, Supplementary Table S1). Similarly, the sediment samples were categorized into the low-salinity-sediment group and the high-salinity-sediment group (Supplementary Figure S2B). The dramatic environmental heterogeneity may account for the differences in archaea community structure between groups.

**Figure 2:**
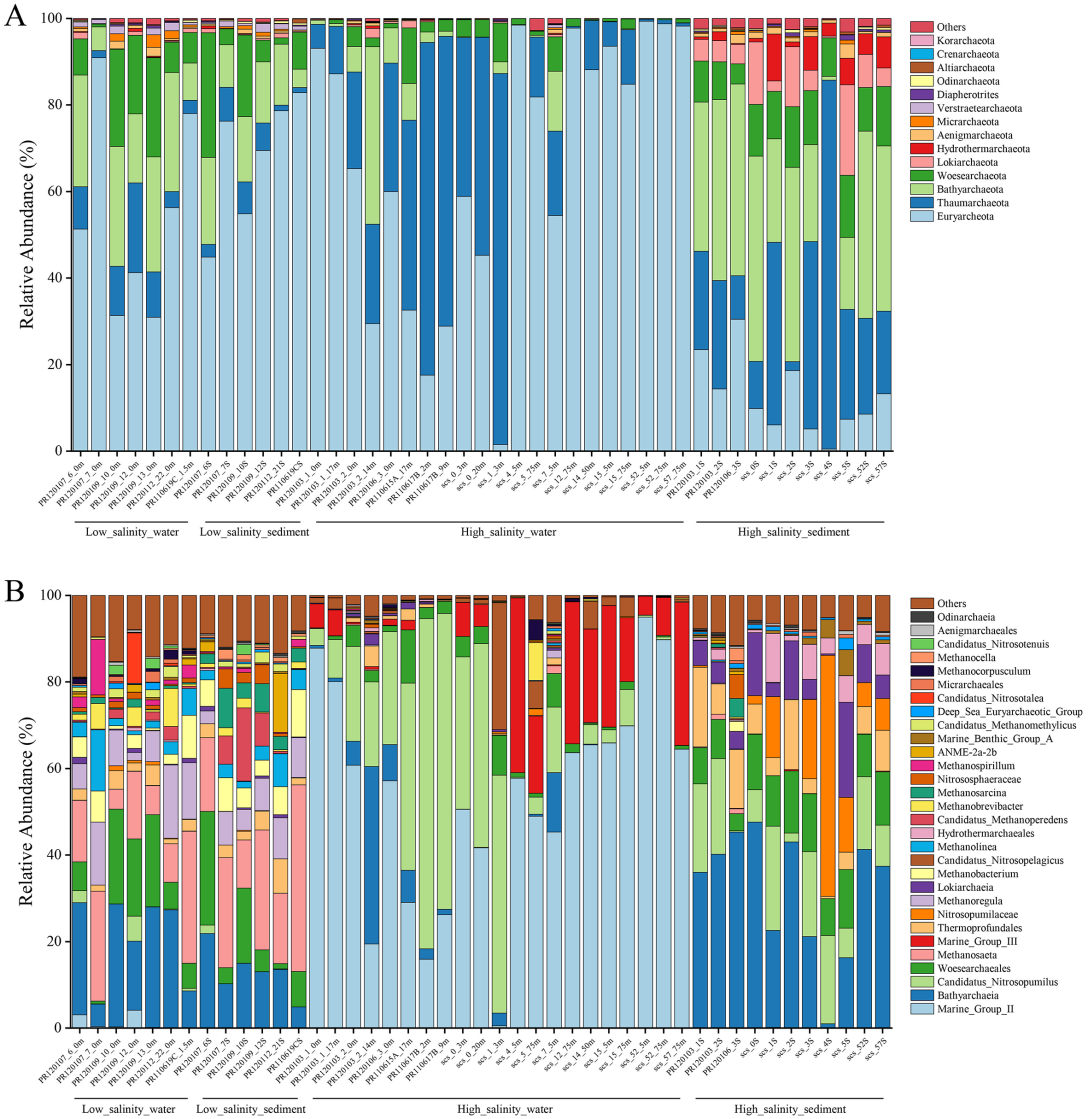
Archaeal community composition at the phylum level **(A)** and genus level **(B)** based on 16S rRNA gene.

The composition of the archaeal communities at the phylum level is shown in Figure 2A. *Euryarchaeota* was the major dominant phylum in almost all low-salinity-water and low-salinity-sediment groups samples. The following were *Bathyarchaeota* and *Woesearchaeota*. *Thaumarchaeota* were also significant components in these two groups of samples. In the high-salinity-water group samples, *Euryarchaeota* and *Thaumarchaeota* were the predominant archaeal phyla. *Bathyarchaeota* and *Woesearchaeota* also account for a certain fraction. Compared with the other three groups, the proportion of *Lokiarchaeota* and *Hydrothermarchaeota* increased in the high-salinity-sediment group samples. *Euryarchaeota*, *Thaumarchaeota*, *Bathyarchaeota*, and *Woesearchaeota* were also dominant components in this group. The community composition of the low-salinity-water and low-salinity-sediment group samples was similar at the genus level (Figure 2B). The dominant Archaea genera were *Bathyarchaeia*, *Woesearchaeales*, *Thermoprofundales, Methanosaeta*, *Methanoregula*, *Methanobacterium*, *Candidatus* Methanoperedens, *Methanolinea*, and *Methanosarcina*. Marine Group II was dominant in most high-salinity-water group samples, except for samples PR120103_2_14 m, PR110615A_17 m, PR110617B_2 m, PR110617B_9 m, SCS_0_20 m, and SCS_1_3 m. *Bathyarchaeia* was dominant in sample PR120103_2_14 m, and *Candidatus* Nitrosopumilus was dominant in the other five samples. Marine Group III had a high proportion in some high-salinity-water group samples. *Bathyarchaeia*, *Candidatus* Nitrosopumilus, *Woesearchaeales*, *Thermoprofundales*, *Nitrosopumilaceae*, *Lokiarchaeia*, and *Hydrothermarchaeales* were dominant genera in the high-salinity-sediment group samples.

After filtering out the AOA OTUs of less than 1% in all samples, the others were mapped to a specific evolutionary tree (Alves et al., 2018) to determine their phylogenetic clades (Supplementary Figure S3, Supplementary Table S2). A total of 121 AOA OTUs were assigned to 20 subgroups, and sediments harbored greater clade diversity than water (Supplementary Figure S4, Supplementary Table S2). NP-α-1 was the dominant *amoA* type in almost all low-salinity-water group samples, except for sample PR110619C_1.5 m, whose community composition was more similar to that of the low-salinity-sediment group samples. In the low-salinity-sediment group samples, NS − δ, NP-η, and NP-γ-2.2 were dominant subgroups, and NT-α had a high abundance in sample PR120112_21S. Sample PR110617B_9 m had a different community composition from the other samples in the high-salinity-water group. In PR110617B_9 m, NP-γ-2.1 had the highest abundance, followed by NS − δ. NP-α-1 and NP-ε were dominant subgroups in the other samples. In the high-salinity-sediment group samples, NP-α-1, NP-α-2.2.4, NP-δ, NP-α-2.1, NP-α-2.2.3, and NP-θ were the predominant subgroups.

Principal component analysis showed that the community exhibited obvious group segregation characteristics. This is in line with the changes of the relative abundance of dominant archaeal clades in each group (Supplementary Figure S5A). This pattern is supported by ANOSIM analysis, where large differences between clusters were observed (Supplementary Table S3). It is worth noting that there was no significant separation between the low-salinity-water and low-salinity-sediment group samples according to the ANOSIM analysis, which was consistent with the PCA. The AOA subcommunities differed significantly among the four groups (Supplementary Table S4). The AOA composition differed significantly between the low-salinity-water and low-salinity-sediment groups (Supplementary Table S4), indicating the specificity of AOA between water and sediments. The differences in AOA composition between the high-salinity-water and low-salinity-water groups were small (Supplementary Table S4), indicating a high tolerance of AOA to salinity.

For each sample, the diversity indices including the Shannon index and the Observed-species number, were shown in Supplementary Table S1. The Shannon and observed-species diversity indices of the high-salinity-water group samples were significantly lower than those of the other three groups, while these indices were not significantly different among the three groups (Supplementary Figure S6A). For AOA subcommunities, the Shannon and observed-species indices of the high-salinity-water and low-salinity-water groups were lower than those of the high-salinity-sediment and low-salinity-sediment groups (Supplementary Figure S6B).

### 3.2. Geographic pattern of archaeal community

**Figure 3:**
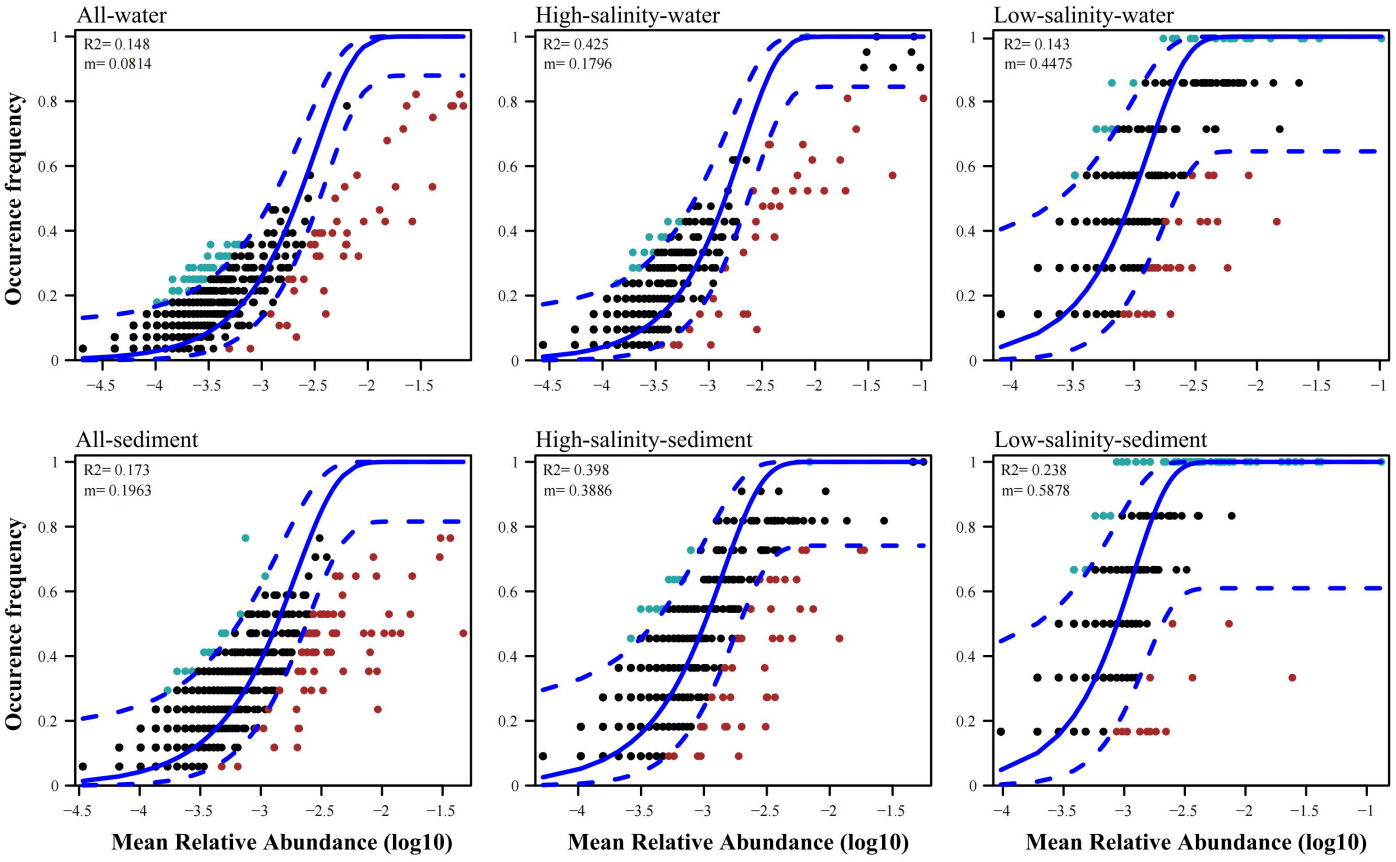
Fit of the neutral community model (NCM) of community assembly for all water, all sediment, the high-salinity-water, low-salinity-water, high-salinity-sediment, and low-salinity-sediment groups samples from the Pearl River to the northern South China sea region. The solid blue lines indicate the best fit to the neutral community model (NCM), and the dashed blue lines represent 95% confidence intervals around the NCM prediction. OTUs that occur more or less frequently than predicted by the NCM are shown in green and red, respectively. m indicates the immigration rate, R^2^ indicates the fit to this model.

The archaeal community immigration rate was estimated to be 0.1796, 0.4475, 0.3886, and 0.5878 in the high-salinity-water, low-salinity-water, high-salinity-sediment, and low-salinity-sediment groups, respectively (Figure 3). In addition, the archaeal community immigration rates across all water and all sediment were smaller than the corresponding high-salinity and low-salinity groups (Figure 3), indicating that the species dispersal within groups was stronger than that among groups.

The high-salinity-water, low-salinity-water, high-salinity-sediment, and low-salinity-sediment groups contained 11, 75, 60, and 73 OTUs, respectively, which occurred in over 70% of their samples (Figure 3). However, only 9 and 9 OTUs occurred in more than 70% of all-water and all-sediment samples (Figure 3), indicating that most archaeal species can only be found in specific groups and few generalists. To judge whether the high occurrence frequency of OTUs was due to differences in the number of samples between groups, we plotted bubble charts for OTUs that occurred in more than 70% of all-water and all-sediment samples. The results showed that only *Ca.* Nitrosopumilus (OTU7 and OTU37) were able to occur in both the high-salinity-water and low-salinity-water group samples (Supplementary Figure S7A), indicating the high tolerance of *Ca.* Nitrosopumilus to salinity. However, MGII and MGIII only appeared in the high-salinity-water group, indicating that they were only adapted to the high-salinity-water environment, and their high occurrence frequency was due to the larger number of samples in the high-salinity-water group. In all sediment samples, MBGD (OTU101) and *Bathyarchaeia* (OTU448 and OTU72) were present in both the high-salinity-sediment and low-salinity-sediment samples (Supplementary Figure S7B). The high frequency of other OTUs was due to the larger number of samples in the high-salinity-sediment group. We randomly selected the same number of high-salinity and low-salinity samples to draw the neutral community model, and the results were consistent with the bubble charts (Supplementary Figure S8), indicating that *Ca.* Nitrosopumilus in water, and MBGD and *Bathyarchaeia* in sediment were archaeal taxa that occurred with high frequency across drastic salinity gradients from the PR to NSCS.

### 3.3. Network analysis of archaeal communities

**Figure 4:**
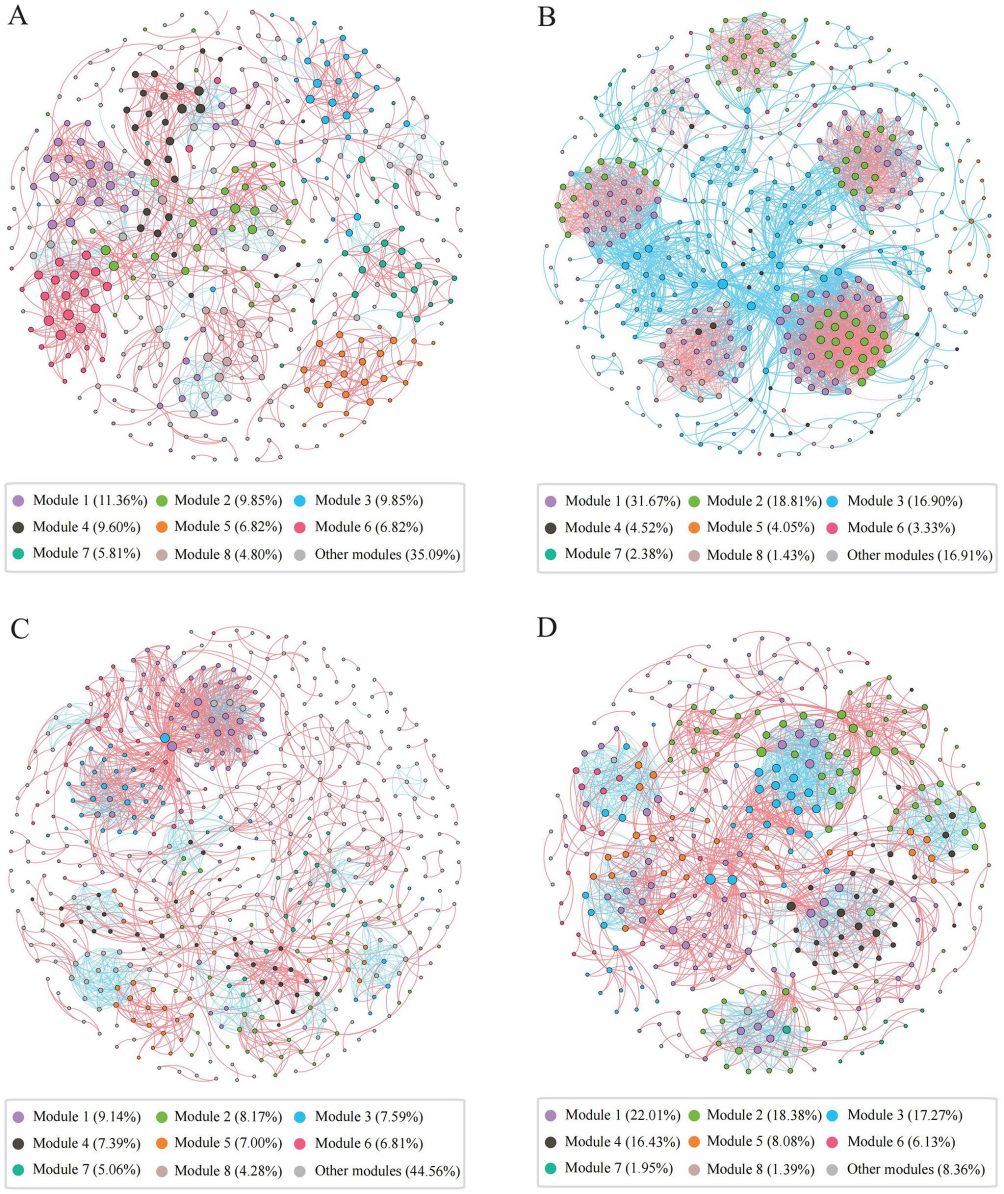
Co-occurrence networks of the archaeal community in the high-salinity-water **(A)**, low-salinity-water **(B)**, high-salinity-sediment **(C)**, and low-salinity-sediment **(D)** groups based on pairwise Spearman’s correlations between OTUs. Each shown connection has a correlation coefficient|*r*| > 0.6 and a *p* value <0.05. The red edges represent significant positive correlations, and the blue edges represent significant negative correlations. The size of each node is proportional to the number of connections, and each node was colored by modularity.

We constructed archaeal co-occurrence networks in water and sediments (Supplementary Figure S9), and analyzed the high-salinity-water, low-salinity-water, high-salinity-sediment, and low-salinity-sediment subnetworks (Figure 4, Supplementary Table S5). The topological properties of these subnetworks varied significantly with salinity. The proportion of negative links in the low-salinity environments was higher than that in the high-salinity environments (Figure 4, Supplementary Table S5), which may indicate that more archaeal taxa in the low-salinity environments tend to adopt a competitive survival style. The eight largest modules accounted for 64.91, 83.09, 55.44, and 91.64% of the high-salinity-water, low-salinity-water, high-salinity-sediment, and low-salinity-sediment subnetworks, respectively (Figure 4). And the network diameter and average path length of the low-salinity groups were lower than those of the high-salinity groups, while the density and average clustering coefficient were higher than those of the high-salinity groups (Supplementary Table S5), indicating that the archaeal community in the low-salinity groups had closer interactions than that in the high-salinity groups. Furthermore, power-law distributions rather than Poisson distributions were observed for all network degrees (Supplementary Table S5). Accordingly, the network presented a structure of scale-free and non-random distribution. The observed network parameters (i.e., average path length, average clustering coefficient, and modularity index) were greater than the corresponding Erdös-Réyni random networks (Supplementary Table S5), indicating a “small world” nature and modular structure.

In the water network, archaeal communities were dominated by taxa that preferred high salinity within modules III and VII, and by taxa that preferred low salinity within modules I, II, IV, V, VI, and VII (Supplementary Figures 9B,C). Furthermore, MGII, MGIII, and *Candidatus* Nitrosopumilus exhibited the highest degrees of centrality in the modules for high salinity levels. In the modules for low salinity levels, however, *Woesearchaeales*, *Bathyarchaeia*, *Methanosaeta*, and *Methanobacterium* exhibited the highest degrees of centrality (Supplementary Figure S9A, Supplementary Table S6). Similarly, in the sediment network, archaeal communities were dominated by taxa preferring high salinity in modules I, IV, V, and VII (Supplementary Figures S9E,F), with *Bathyarchaeia*, *Woesearchaeales*, and *Nitrosopumilaceae* exhibiting the highest degrees of centrality (Supplementary Figure S9D, Supplementary Table S7); while archaeal communities were dominated by taxa preferring low salinity in modules II, III, VI, and VII. *Bathyarchaeia*, *Woesearchaeales*, and *Methanosaeta* exhibited the highest degrees of centrality. Finally, we compared the network stability of different archaeal subnetworks. The low-salinity subnetworks had higher natural connectivity than the high-salinity subnetworks (Supplementary Figure S10), indicating that the low-salinity subnetworks had stronger robustness.

### 3.4. Ecological processes of archaeal community assembly

**Figure 5:**
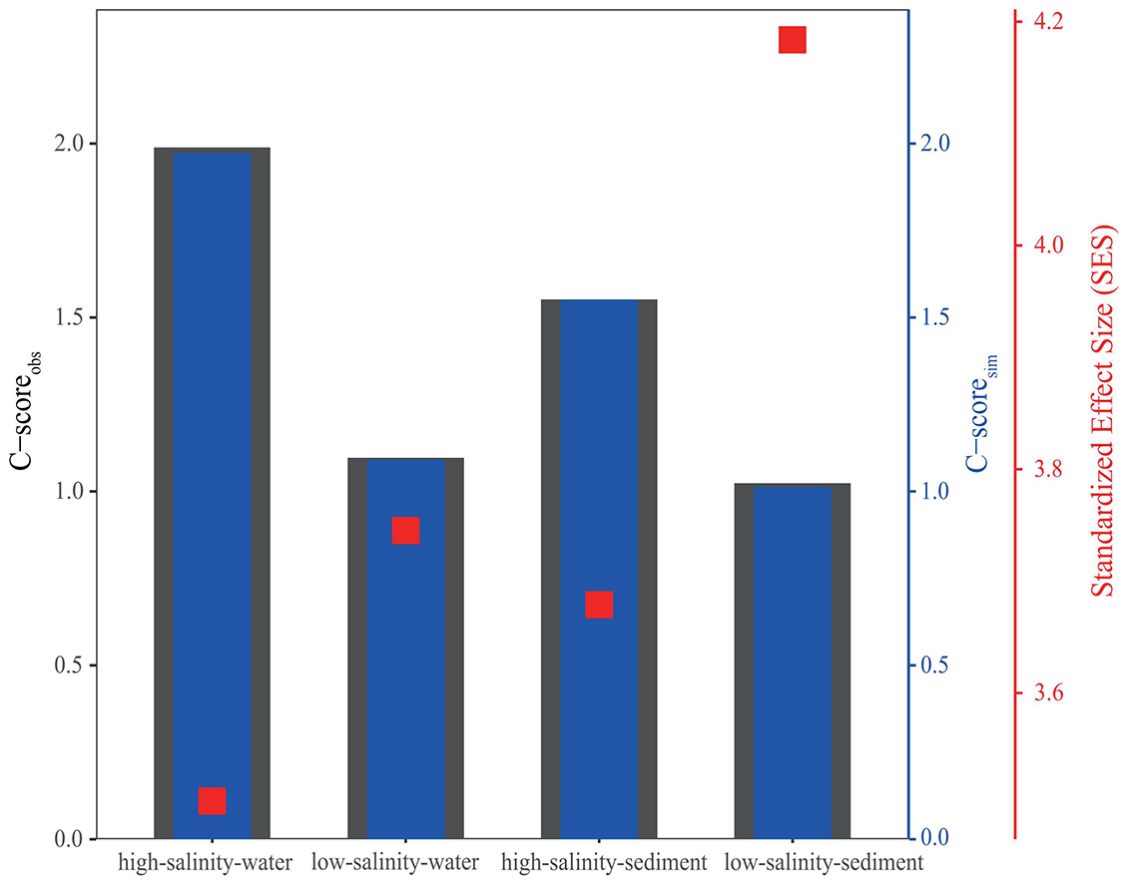
C-score metric using null models. The values of observed C-score (C-score_obs_) > simulated C-score (C-score_sim_) indicate non-random co-occurrence patterns. Standardized effect size (SES) < −2 and > 2 represents aggregation and segregation, respectively.

The standardized effect size (SES) was calculated based on OTU abundance to investigate the relative contributions of deterministic and stochastic processes to archaeal community assembly (Figure 5). The SES values in all four groups were greater than 2, the threshold for stochastic and deterministic processes, indicating that deterministic processes contributed more to archaeal community assembly than stochastic processes from the PR to the NSCS (Figure 5). Consistent with this, the relationship between OTU occurrence frequency and relative abundance can only be characterized by the neutral community model to a small extent (Figure 3), indicating that stochastic processes accounted for a smaller proportion of archaeal community assembly. For the high-salinity-sediment, low-salinity-sediment, high-salinity-water, and low-salinity-water groups, stochastic processes explained 39.8, 23.8, 42.5, and 14.3% of the community variation, respectively, showing a trend of decreasing gradually with the decrease in salinity. This indicated that stochastic processes contributed more in the high-salinity than low-salinity environments from the PR to the NSCS. Furthermore, stochastic processes showed lower contributions to archaeal community assembly in all-water and all-sediment samples (Figure 3). Additionally, in the neutral community model with the same number of high-salinity and low-salinity samples, the results suggested that stochastic processes contributed less to the assembly of archaeal community. In planktonic archaeal communities, the neutral community model (NCM) cannot even describe the archaeal community composition (Supplementary Figure S8). All archaeal communities had wider niche breadths at the high salinity than at the low salinity levels (Supplementary Table S8).

## 4. Discussion

### 4.1. Heterogeneity of archaeal community structure from the PR to the NSCS

This study revealed detailed archaeal community composition across four environments from the PR to the NSCS by high-throughput sequencing analysis. Although the archaeal communities were sampled across time and seasons, ANOSIM analysis revealed that archaeal communities differed significantly among habitats in the current study (Supplementary Table S3). Because the archaeal community samples in the four groups were segregated in PCA (Supplementary Figure S5A), we surmised that seasonal differences within groups were unlikely to be more influential than differences among groups in this study. This result confirmed previous findings that benthic communities of bacterial (Böer et al., 2009) and microbial eukaryotes (Gong et al., 2015) varied with sediment depth or area rather than the season.

Archaeal community structure showed a distinct pattern of heterogeneity (Figure 2). Previous reports have indicated that the structure of microbial communities varies spatially from estuaries to the open ocean (Herlemann et al., 2011; Liu et al., 2014). It can also be seen here that the composition of planktonic and benthic archaeal communities differed considerably from the PR to the NSCS. We observed a high abundance of methanogenic archaea in the low-salinity environments (Figure 2B). Salinity is reportedly the most important abiotic factor governing the distribution of methanogenic communities worldwide and determines which lineages may survive to a large extent (Wen et al., 2017). Increasing salinity is reported to restrain hydrogenotrophic methanogens while promoting acetoclastic methanogenesis (Liu et al., 2016). Additionally, the activity of methanogens that utilize acetate or H_2_ was highly impacted by pH. Low pH, for example, makes acetoclastic methanogenesis difficult because it lowers acetate dissociation (Megonigal et al., 2004; Kotsyurbenko et al., 2007). MGII and MGIII were typically found in seawater and were almost absent in freshwater environments, suggesting that suitable salinity is necessary for their survival. *Bathyarchaeota* were widespread in the low-salinity environments and the high-salinity-sediment samples, and were the dominant taxa in these environments (Figure 2). As one of the most important and active microbial groups in freshwater and marine sediments, the relative abundance of *Bathyarchaeota* accounted for 36 ± 22% of the total archaea abundance (Fillol et al., 2016). Previous studies have shown that different *Bathyarchaeota* subgroups are indicators for different environments: subgroups 1 and 8 in marine environments, and subgroups 5 and 11 in freshwater environments (Fillol et al., 2016; Wang et al., 2020).

*Thaumarchaeota* was suggested to be a salinity-tolerant species (Xie et al., 2014). Based on *amoA* genotypes suggested by Alves et al. (2018), we determined the AOA subcommunity composition from the PR to the NSCS in the current study (Supplementary Figure S4). NS-δ is abundant in the low-salinity sediment samples. Previous studies have indicated that clade NS-δ accounts for a significant proportion of AOA communities in salt lakes and freshwater habitats worldwide (Alves et al., 2018). Considering its global environmental distribution, NS-δ represents either versatile microorganisms or ubiquitous soil microorganisms that are frequently transferred to adjacent rivers. A systematic analysis of AOA on a global scale found that clades NP-α, NP-ε, NP-θ, and NP-ζ occur primarily in marine environments, NP-γ occurs primarily in freshwater and estuarine-coastal environments, and NP-η occurs more frequently in freshwater and soils-sediments (Alves et al., 2018), consistent with the results in the low-salinity environments in the current study (Supplementary Figure S4). The composition of AOA clades in seawater and sediment is almost different: most AOA in seawater belong to clades NP-ε-2 and NP-α-2.2.2.1, while sediment harbor greater clade diversity, including NP-δ, NP-γ, NP-θ, and NP-α (Durbin and Teske, 2010; Alves et al., 2018; Zou et al., 2020a), which is consistent with the results in the high-salinity environments presented here (Supplementary Figure S4). Overall, our results showed that the change in environmental conditions from the PR to the NSCS controlled the distribution of archaeal communities.

### 4.2. Significant differences in the geographic pattern of archaeal communities from the PR to the NSCS

The archaeal community immigration rates in the low-salinity environments were significantly higher than those in the high-salinity environments (Figure 3), suggesting the higher dispersal ability of most archaeal taxa in the upper PR than in the NSCS. These results may be attributed to the faster flow in the upper PRE, which facilitated the dispersal of archaeal communities in the water and surface sediment. Additionally, the species dispersal of archaea among groups was smaller than that within groups, indicating that the majority of archaea had difficulty surviving across drastic salinity gradients. This is consistent with the results of the neutral community model, in which the majority of archaea only occurred in specific groups. Only *Ca.* Nitrosopumilus (OTU7 and OTU37) in water, and *Bathyarchaeia* (OTU448 and OTU72) in sediments were able to occur in both low-salinity and high-salinity environments. Previous studies have shown that salinity is an important environmental factor limiting the distribution of archaea (Webster et al., 2015), *Thaumarchaeota* was suggested to be a salinity-tolerant species (Xie et al., 2014), and *Bathyarchaeia* is widely present in various environments with high phylogenetic diversity and abundance (Fillol et al., 2016; Wang et al., 2020).

### 4.3. Co-occurrence network patterns and keystone taxa in archaeal community from the PR to the NSCS

Rather than growing in isolation, microbes in natural ecosystems prioritize forming complex interaction networks (Banerjee et al., 2019). Both the composition and dynamics of microbial communities are strongly influenced by microbial interactions, and co-occurrence networks can reveal interactions between species, such as competition and cooperation (Berry and Widder, 2014; Lima-Mendez et al., 2015; Wei et al., 2016). The mostly positive correlations in the high-salinity subnetworks identified in this study (Figure 4, Supplementary Table S5) suggest that cooperation is more prevalent than competition in archaeal communities (Berry and Widder, 2014). While this phenomenon is common in natural ecosystems, it is perhaps not surprising since many microbes rely heavily on cross-feeding, co-aggregation, co-colonization, or niche overlap and construction (Faust and Raes, 2012; Faust et al., 2015; Shi et al., 2019). A previous study that focused on the co-occurrence networks under the influence of graphene oxide and different temperatures found that when facing the enhancement of heterogeneity selection, the strategy of the bacterial community was to enhance the positive correlation and shared niche, while the strategy of the archaeal community was to enhance the negative correlation and competition (Liao et al., 2022), which is consistent with more negative correlations in low-salinity environments with higher environmental heterogeneity (Figure 4).

Our results showed that network modules corresponded well to groups (Supplementary Figure S9). This suggested that the modular structure or properties of planktonic and benthic archaeal communities were sensitive to changes in environmental conditions. Studies have shown that salinity and other environmental factors (i.e., pH, temperature, and nutrients) could change bacterial and eukaryotic plankton co-occurrence networks (Ji et al., 2019; Liu et al., 2019). Environmental heterogeneity can induce microbial modularity (Röttjers and Faust, 2018), explaining why these modules predominated across different groups. Modularity could be used to indicate both niche differentiation and competitive/cooperative relationships, leading to non-random network structures that ultimately increase the ecological network complexity (Olesen et al., 2007).

For low-salinity subnetworks, the average clustering coefficient in the network topology parameters was higher, implying the higher complexity of these subnetworks. Highly complex networks tend to be more stable due to network buffering (Landi et al., 2018), so the low-salinity subnetworks may be more stable, while the high-salinity subnetworks may be less stable, which was also supported by Supplementary Figure S10. This pattern may be closely related to archaeal community diversity and richness, as higher diversity and richness may lead to more complex network structures (Chen and Wen, 2021). Changes in environmental heterogeneity may be the most important factor responsible for different patterns of microbial interactions among groups, as the complexity and connectivity of networks often had positive correlations with environmental heterogeneity (Mougi and Kondoh, 2012). Along the upper reaches of the Pearl River to the estuary (low-salinity groups), fresh water with low salinity and high-concentration nutrients and organic matter characteristics meets seawater with high salinity and low-concentration nutrients and organic matter, to form a highly environmental heterogeneity. And this heterogeneity decreased along the estuary to the shelf (high-salinity groups) (Supplementary Figure S12). Microbial communities were more likely to aggregate and form more connected networks in more heterogeneous environments (Morriën et al., 2017; Li et al., 2019). Conversely, in more homogeneous environments, microbial communities tended to occupy similar biological niches, resulting in fewer interactions (Cherif and Loreau, 2007; Freilich et al., 2011; Lu et al., 2022).

Since nodes with a higher degree of centrality are more likely to be important to preserve taxa coexistence, MGII, MGIII, and *Candidatus* Nitrosopumilus may play a key role in maintaining taxa coexistence in the high-salinity-water subnetwork, while *Woesearchaeales*, *Bathyarchaeia*, *Methanosaeta*, and *Methanobacterium* are more important in maintaining coexistence in the low-salinity-water subnetwork (Supplementary Table S6). Similarly, in the high-salinity-sediment subnetwork, *Bathyarchaeia*, *Woesearchaeales*, and *Nitrosopumilaceae* may play a key role in maintaining taxa coexistence, while *Bathyarchaeia*, *Woesearchaeales*, and *Methanosaeta* are more important in maintaining coexistence in the low-salinity-sediment subnetwork (Supplementary Table S7).

### 4.4. Archaeal community assembly mainly shaped by deterministic processes from the PR to the NSCS

The C-score analysis supported that deterministic processes played a relatively more important role than stochastic processes, whether in the water or sediments from the PR to the NSCS (Figure 5). Previous studies have indicated that geographic scales and environmental gradients largely explain the balance between deterministic and stochastic processes (Hanson et al., 2012). In general, at relatively large spatial scales (~12,000 km), deterministic (environmental) factors have been found to exert a greater impact on benthic microbial communities than stochastic (spatial) factors (Zhang et al., 2018), while at smaller spatial scales (~20 to 1,500 km), the contribution of stochastic processes was greater (Chen et al., 2017; Liu et al., 2020; Lu et al., 2022). However, in the current study with a small spatial scale (~600 km), drastic changes in environmental factors were found in both low-salinity and high-salinity environments, resulting in a greater contribution of deterministic processes than stochastic processes at these sites, which may imply the importance of environmental factors for the construction of archaeal communities from the PR to the NSCS (Xie et al., 2014, 2018; Zou et al., 2020b). Consistent with our results, a previous study found that deterministic processes had a stronger effect on soil bacterial communities in a 1,092 km range, which was mainly driven by a large pH gradient (Shi et al., 2018). In a study about the prokaryote community assembly from three parallel ~100 km transition sections in the lower Pearl River Estuary, a greater role of stochasticity to the benthic prokaryote community assembly was observed (Lu et al., 2022). However, it should be noted that the relative contribution of stochastic processes to the microbial community assembly was only 54.02%, which may be because the objects of this study were prokaryotes, and the smaller spatial scale led to less environmental heterogeneity compared with our study. Additionally, the C-score revealed that the SES values increased with decreasing salinity, implying the enhanced importance of deterministic processes in archaeal communities, showing the relative contribution of deterministic processes to low-salinity samples was higher than that of high-salinity samples, possibly due to more heterogeneous environment attributed to the steeper environmental gradients in the low-salinity environments (Supplementary Figure S12), as well as the more spatially inconsistent and complex hydrologic movement (Stegen et al., 2015).

The high-salinity environments had wider archaeal community niche breadths, while the low-salinity environments had narrower community niche breadths (Supplementary Table S8), indicating that the high-salinity environments had more generalists that can adapt to a wide range of environmental niches, while the low-salinity environments had more specialists. This result may be attributed to the higher nutrient and organic matter content in the low-salinity environments, which promoted the specialization of the archaeal niche, resulting in narrower habitat niche breadths. Conversely, in the high-salinity environments, the limited availability of resources induced the generalization of the archaeal niche, leading to wider habitat niche breadths (Pandit et al., 2009). A balance between determinism and stochasticity is determined by the resistance of microorganisms to environmental changes, along with spatial scales and gradients in the environment (Liu et al., 2020). Microorganisms that adapt to high-salinity environments have evolved “salt in” and “salt out” strategies to regulate the cytoplasm to osmotic pressure (Oren, 2011). In high-salinity environments with less environmental heterogeneity or fewer competitive interactions among environmental generalists, the relative contribution of stochastic processes may increase (Jiao et al., 2020). Seasonal changes in pollutant concentrations brought about by terrigenous input may put archaea under great selection pressure from the upper Pearl River to the estuary (Yin, 2002; Chen et al., 2009; Lu et al., 2018). The results of the neutral community models indicated that the stochastic processes had a low explanation for the archaeal community composition from the PR to the NSCS (Figure 3) or even could not explain it (Supplementary Figure S8). The difference in the explanation of the archaeal community between the two neutral community models may be due to more samples in the high-salinity groups, which increased the proportion of stochastic processes. Additionally, the archaeal communities exhibited narrower niches in the low-salinity environments than that in the high-salinity environments (Supplementary Table S8), implying that the assembly of archaeal communities under low-salinity conditions was more affected by deterministic processes, possibly because deterministic processes generally have greater effects on habitat specialists with narrow niche breadths than on generalists with wide niche breadths (Pandit et al., 2009; Wu et al., 2018).

Although the C-score analysis and NCM well described the archaeal community assembly patterns in the current study, it is difficult to infer the specific effects of environmental and spatial variables on archaeal community structure (Chisholm and Pacala, 2010). In addition, the impact of unmeasured environmental factors and species interactions on archaeal community structure needs to be considered (Vellend et al., 2014; Zhou and Ning, 2017). Because estuarine ecosystems are overly dynamic, some key environmental factors may change in a stochastic manner, and our sampling involved different seasons, which may mask the main ecological patterns. To fully understand the archaeal community assembly mechanisms in subtropical estuaries, we need further experimental programs that take space and time into consideration.

## 5. Conclusion

Our work showed the majority of archaea had difficulty surviving across drastic salinity gradients, with only *Thaumarchaeota* in water and *Bathyarchaeota* in sediments able to occur in both low-salinity and high-salinity environments. The species dispersal of archaea was stronger in low-salinity than high-salinity environments. Low-salinity environments exhibited stronger environmental heterogeneity, which imposed intense selective pressure on archaeal communities, resulting in deterministic processes dominating the assembly of archaeal communities, while archaea enhanced community stability through competition, adapting to the severe environmental fluctuations.

## Data availability statement

The datasets presented in this study can be found in online repositories. The names of the repository/repositories and accession number(s) can be found below: https://www.ncbi.nlm.nih.gov/, PRJNA824672. https://www.ncbi.nlm.nih.gov/, PRJNA825103.

## Author contributions

XL conducted the data analysis and wrote the manuscript. WX devised the project, conducted the experiments, data analysis, and revised the manuscript. CZ devised the project and revised the manuscript. All authors contributed to the article and approved the submitted version.

## Funding

This work was financially supported by the State Key R&D project of China grant (No. 2018YFA0605800); the National Natural Science Foundation of China (Nos. 92051117, 41776137, 91851210, 42141003); the Shenzhen Key Laboratory of Marine Archaea Geo-Omics, Southern University of Science and Technology (No. ZDSYS201802081843490), the Southern Marine Science and Engineering Guangdong Laboratory (Guangzhou) (No. K19313901), and Guangdong Basic and Applied Basic Research Foundation (No. 2021B1515120080).

## Conflict of interest

The authors declare that the research was conducted in the absence of any commercial or financial relationships that could be construed as a potential conflict of interest.

## Publisher’s note

All claims expressed in this article are solely those of the authors and do not necessarily represent those of their affiliated organizations, or those of the publisher, the editors and the reviewers. Any product that may be evaluated in this article, or claim that may be made by its manufacturer, is not guaranteed or endorsed by the publisher.

## References

[ref1] Ait Alla A. Mouneyrac C. Durou C. Moukrim A. Pellerin J. (2006). Tolerance and biomarkers as useful tools for assessing environmental quality in the Oued Souss estuary (bay of Agadir, Morocco). Comp. Biochem. Physiol. Toxicol. Pharmacol. 143, 23–29. doi: 10.1016/j.cbpc.2005.11.015, PMID: 16413830

[ref2] Alves R. J. E. Minh B. Q. Urich T. von Haeseler A. Schleper C. (2018). Unifying the global phylogeny and environmental distribution of ammonia-oxidising archaea based on amoA genes. Nat. Commun. 9:1517. doi: 10.1038/s41467-018-03861-1, PMID: 29666365 PMC5904100

[ref3] Bahram M. Kohout P. Anslan S. Harend H. Abarenkov K. Tedersoo L. (2016). Stochastic distribution of small soil eukaryotes resulting from high dispersal and drift in a local environment. ISME J. 10, 885–896. doi: 10.1038/ismej.2015.164, PMID: 26394006 PMC4796928

[ref4] Baird D. Christian R. R. Peterson C. H. Johnson G. A. (2004). Consequences of hypoxia on estuarine ecosystem function: energy diversion from consumers to microbes. Ecol. Appl. 14, 805–822. doi: 10.1890/02-5094

[ref5] Banerjee S. Walder F. Büchi L. Meyer M. Held A. Y. Gattinger A.. (2019). Agricultural intensification reduces microbial network complexity and the abundance of keystone taxa in roots. ISME J. 13, 1722–1736. doi: 10.1038/s41396-019-0383-2, PMID: 30850707 PMC6591126

[ref6] Barbera P. Kozlov A. M. Czech L. Morel B. Darriba D. Flouri T.. (2019). EPA-ng: massively parallel evolutionary placement of genetic sequences. Syst. Biol. 68, 365–369. doi: 10.1093/sysbio/syy054, PMID: 30165689 PMC6368480

[ref7] Bastian M. Heymann S. Jacomy M. (2009). Gephi: an open source software for exploring and manipulating networks, Proceedings of the International AAAI Conference on Web and Social Media, 361–362.

[ref8] Bernhard A. E. Bollmann A. (2010). Estuarine nitrifiers: new players, patterns and processes. Estuar. Coast. Shelf Sci. 88, 1–11. doi: 10.1016/j.ecss.2010.01.023

[ref9] Berry D. Widder S. (2014). Deciphering microbial interactions and detecting keystone species with co-occurrence networks. Front. Microbiol. 5:e00219. doi: 10.3389/fmicb.2014.00219, PMID: 24904535 PMC4033041

[ref10] Böer S. I. Hedtkamp S. I. Van Beusekom J. E. Fuhrman J. A. Boetius A. Ramette A. (2009). Time- and sediment depth-related variations in bacterial diversity and community structure in subtidal sands. ISME J. 3, 780–791. doi: 10.1038/ismej.2009.29, PMID: 19340087

[ref11] Bolyen E. Rideout J. R. Dillon M. R. Bokulich N. A. Abnet C. C. Al-Ghalith G. A.. (2019). Reproducible, interactive, scalable and extensible microbiome data science using QIIME 2. Nat. Biotechnol. 37, 852–857. doi: 10.1038/s41587-019-0209-9, PMID: 31341288 PMC7015180

[ref12] Canfield D. E. Thamdrup B. (2009). Towards a consistent classification scheme for geochemical environments, or, why we wish the term ‘suboxic’would go away. Geobiology 7, 385–392. doi: 10.1111/j.1472-4669.2009.00214.x, PMID: 19702823

[ref13] Chen B. Liu H. Landry M. R. Chen M. Sun J. Shek L.. (2009). Estuarine nutrient loading affects phytoplankton growth and microzooplankton grazing at two contrasting sites in Hong Kong coastal waters. Mar. Ecol. Prog. Ser. 379, 77–90. doi: 10.3354/meps07888

[ref14] Chen W. Pan Y. Yu L. Yang J. Zhang W. (2017). Patterns and processes in marine microeukaryotic community biogeography from Xiamen coastal waters and intertidal sediments, Southeast China. Front. Microbiol. 8:1912. doi: 10.3389/fmicb.2017.01912, PMID: 29075237 PMC5644358

[ref15] Chen W. Ren K. Isabwe A. Chen H. Liu M. Yang J. (2019). Stochastic processes shape microeukaryotic community assembly in a subtropical river across wet and dry seasons. Microbiome 7, 1–16. doi: 10.1186/s40168-019-0763-x, PMID: 31640783 PMC6806580

[ref16] Chen W. Wen D. (2021). Archaeal and bacterial communities assembly and co-occurrence networks in subtropical mangrove sediments under *Spartina alterniflora* invasion. Environ. Microb. 16, 10–18. doi: 10.1186/s40793-021-00377-y, PMID: 33941277 PMC8091715

[ref17] Cherif M. Loreau M. (2007). Stoichiometric constraints on resource use, competitive interactions, and elemental cycling in microbial decomposers. Am. Nat. 169, 709–724. doi: 10.1086/516844, PMID: 17479458

[ref18] Chisholm R. A. Pacala S. W. (2010). Niche and neutral models predict asymptotically equivalent species abundance distributions in high-diversity ecological communities. Proc. Natl. Acad. Sci. U. S. A. 107, 15821–15825. doi: 10.1073/pnas.1009387107, PMID: 20733073 PMC2936647

[ref19] Cloern J. Foster S. Kleckner A. (2014). Phytoplankton primary production in the world's estuarine-coastal ecosystems. Biogeosciences 11, 2477–2501. doi: 10.5194/bg-11-2477-2014

[ref20] Crump B. Peterson B. Raymond P. Amon R. M. W. Rinehart A. McClelland J.. (2009). Circumpolar synchrony in big river bacterioplankton. Proc. Natl. Acad. Sci. U. S. A. 106, 21208–21212. doi: 10.1073/pnas.0906149106, PMID: 19940248 PMC2783008

[ref21] Durbin A. M. Teske A. (2010). Sediment-associated microdiversity within the marine group I Crenarchaeota. Environ. Microbiol. Rep. 2, 693–703. doi: 10.1111/j.1758-2229.2010.00163.x, PMID: 23766257

[ref22] Elzhov T. V. Mullen K. M. Spiess A. N. Bolker B. (2016). Minpack. Lm: R interface to the Levenberg-Marquardt nonlinear least-squares algorithm found in MINPACK, plus support for bounds. R package version 1.2–1. R-project. Org/package= minpack. Lm.

[ref23] Erdos P. Rényi A. (1960). On the evolution of random graphs. Publ. Math. Inst. Hung. Acad. Sci. 5, 17–60.

[ref24] Faust K. Lima-Mendez G. Lerat J.-S. Sathirapongsasuti J. F. Knight R. Huttenhower C.. (2015). Cross-biome comparison of microbial association networks. Front. Microbiol. 6:1200. doi: 10.3389/fmicb.2015.01200, PMID: 26579106 PMC4621437

[ref25] Faust K. Raes J. (2012). Microbial interactions: from networks to models. Nat. Rev. Microbiol. 10, 538–550. doi: 10.1038/nrmicro2832 22796884

[ref26] Fillol M. Auguet J.-C. Casamayor E. O. Borrego C. M. (2016). Insights in the ecology and evolutionary history of the miscellaneous Crenarchaeotic group lineage. ISME J. 10, 665–677. doi: 10.1038/ismej.2015.143, PMID: 26284443 PMC4817671

[ref27] Francis C. A. Roberts K. J. Beman J. M. Santoro A. E. Oakley B. B. (2005). Ubiquity and diversity of ammonia-oxidizing archaea in water columns and sediments of the ocean. Proc. Natl. Acad. Sci. U. S. A. 102, 14683–14688. doi: 10.1073/pnas.0506625102, PMID: 16186488 PMC1253578

[ref28] Freilich S. Zarecki R. Eilam O. Segal E. S. Henry C. S. Kupiec M.. (2011). Competitive and cooperative metabolic interactions in bacterial communities. Nat. Commun. 2, 1–7. doi: 10.1038/ncomms1597 22158444

[ref29] Gantner S. Andersson A. F. Alonso-Sáez L. Bertilsson S. (2011). Novel primers for 16S rRNA-based archaeal community analyses in environmental samples. J. Microbiol. Methods 84, 12–18. doi: 10.1016/j.mimet.2010.10.001, PMID: 20940022

[ref30] Gao G.-F. Peng D. Wu D. Zhang Y. Chu H. (2021). Increasing inundation frequencies enhance the stochastic process and network complexity of the soil archaeal community in coastal wetlands. Appl. Environ. Microbiol. 87, e02560–e02520. doi: 10.1128/AEM.02560-20, PMID: 33741614 PMC8208137

[ref31] Gong J. Shi F. Ma B. Dong J. Pachiadaki M. Zhang X.. (2015). Depth shapes α-and β-diversities of microbial eukaryotes in surficial sediments of coastal ecosystems. Environ. Microbiol. 17, 3722–3737. doi: 10.1111/1462-2920.12763, PMID: 25581721

[ref32] Gotelli N. McCabe D. (2002). Species co-occurrence: a meta-analysis of J. M. Diamond's assembly rules model. Ecology 83, 2091–2096. doi: 10.2307/3072040

[ref33] Hanson C. A. Fuhrman J. A. Horner-Devine M. C. Martiny J. B. (2012). Beyond biogeographic patterns: processes shaping the microbial landscape. Nat. Rev. Microbiol. 10, 497–506. doi: 10.1038/nrmicro2795, PMID: 22580365

[ref34] Harrison P. J. Yin K. Lee J. H. W. Gan J. Liu H. (2008). Physical–biological coupling in the Pearl River estuary. Cont. Shelf Res. 28, 1405–1415. doi: 10.1016/j.csr.2007.02.011

[ref35] Herlemann D. P. Labrenz M. Jürgens K. Bertilsson S. Waniek J. J. Andersson A. F. (2011). Transitions in bacterial communities along the 2000 km salinity gradient of the Baltic Sea. ISME J. 5, 1571–1579. doi: 10.1038/ismej.2011.41, PMID: 21472016 PMC3176514

[ref36] Huang X. Huang L. Yue W. (2003). The characteristics of nutrients and eutrophication in the Pearl River estuary South China. Mar. Poll. Bull. 47, 30–36. doi: 10.1016/S0025-326X(02)00474-5, PMID: 12787594

[ref37] Huber P. Metz S. Unrein F. Mayora G. Sarmento H. Devercelli M. (2020). Environmental heterogeneity determines the ecological processes that govern bacterial metacommunity assembly in a floodplain river system. ISME J. 14, 2951–2966. doi: 10.1038/s41396-020-0723-2, PMID: 32719401 PMC7784992

[ref38] Ji M. Kong W. Yue L. Wang J. Deng Y. Zhu L. (2019). Salinity reduces bacterial diversity, but increases network complexity in Tibetan plateau lakes. FEMS Microbiol. Ecol. 95:fiz190. doi: 10.1093/femsec/fiz190, PMID: 31778180

[ref39] Jiao S. Yang Y. Xu Y. Zhang J. Lu Y. (2020). Balance between community assembly processes mediates species coexistence in agricultural soil microbiomes across eastern China. ISME J. 14, 202–216. doi: 10.1038/s41396-019-0522-9, PMID: 31611655 PMC6908645

[ref40] Katoh K. Standley D. M. (2013). MAFFT multiple sequence alignment software version 7: improvements in performance and usability. Mol. Biol. Evol. 30, 772–780. doi: 10.1093/molbev/mst010, PMID: 23329690 PMC3603318

[ref41] Könneke M. Bernhard A. E. de La Torre J. R. Walker C. B. Waterbury J. B. Stahl D. A. (2005). Isolation of an autotrophic ammonia-oxidizing marine archaeon. Nature 437, 543–546. doi: 10.1038/nature03911, PMID: 16177789

[ref42] Kotsyurbenko O. Friedrich M. Simankova M. Nozhevnikova A. Golyshin P. Timmis K.. (2007). Shift from acetoclastic to H2-dependent methanogenesis in a west Siberian peat bog at low pH values and isolation of an acidophilic Methanobacterium strain. Appl. Environ. Microbiol. 73, 2344–2348. doi: 10.1128/AEM.02413-06, PMID: 17277200 PMC1855669

[ref43] Landi P. Minoarivelo H. Brännström Å. Hui C. Dieckmann U. (2018). Complexity and stability of ecological networks: a review of the theory. Popul. Ecol. 60, 319–345. doi: 10.1007/s10144-018-0628-3

[ref44] Letunic I. Bork P. (2021). Interactive tree of life (iTOL) v5: an online tool for phylogenetic tree display and annotation. Nucleic Acids Res. 49, W293–W296. doi: 10.1093/nar/gkab301, PMID: 33885785 PMC8265157

[ref45] Li Y. Gao Y. Zhang W. Wang C. Wang P. Niu L.. (2019). Homogeneous selection dominates the microbial community assembly in the sediment of the three gorges reservoir. Sci. Total Environ. 690, 50–60. doi: 10.1016/j.scitotenv.2019.07.014, PMID: 31284194

[ref46] Liao Y. Jiang Z. Li S. Dang Z. Zhu X. Ji G. (2022). Archaeal and bacterial ecological strategies in sediment denitrification under the influence of graphene oxide and different temperatures. Sci. Total Environ. 838:156549. doi: 10.1016/j.scitotenv.2022.156549, PMID: 35688242

[ref47] Lima-Mendez G. Faust K. Henry N. Decelle J. Colin S. Carcillo F.. (2015). Determinants of community structure in the global plankton interactome. Science 348:1262073. doi: 10.1126/science.1262073, PMID: 25999517

[ref48] Liu L. Chen H. Liu M. Yang J. R. Xiao P. Wilkinson D. M.. (2019). Response of the eukaryotic plankton community to the cyanobacterial biomass cycle over 6 years in two subtropical reservoirs. ISME J. 13, 2196–2208. doi: 10.1038/s41396-019-0417-9, PMID: 31053831 PMC6776060

[ref49] Liu X. Pan J. Liu Y. Li M. Gu J.-D. (2018). Diversity and distribution of Archaea in global estuarine ecosystems. Sci. Total Environ. 637-638, 349–358. doi: 10.1016/j.scitotenv.2018.05.016, PMID: 29753224

[ref50] Liu Y. Priscu J. Conrad R. Vick-Majors T. Chu H. Hou J. (2016). Salinity drives archaeal distribution patterns in high altitude lake sediments on the Tibetan plateau. FEMS Microbiol. Ecol. 92:fiw033. doi: 10.1093/femsec/fiw033, PMID: 26887660

[ref51] Liu L. Yang J. Yu Z. Wilkinson D. M. (2015). The biogeography of abundant and rare bacterioplankton in the lakes and reservoirs of China. ISME J. 9, 2068–2077. doi: 10.1038/ismej.2015.29, PMID: 25748371 PMC4542038

[ref52] Liu J. Yang H. Zhao M. Zhang X.-H. (2014). Spatial distribution patterns of benthic microbial communities along the pearl estuary China. Syst. Appl. Microbiol. 37, 578–589. doi: 10.1016/j.syapm.2014.10.005, PMID: 25467555

[ref53] Liu J. Zhu S. Liu X. Yao P. Ge T. Zhang X.-H. (2020). Spatiotemporal dynamics of the archaeal community in coastal sediments: assembly process and co-occurrence relationship. ISME J. 14, 1463–1478. doi: 10.1038/s41396-020-0621-7, PMID: 32132664 PMC7242467

[ref54] Lloyd K. G. Schreiber L. Petersen D. G. Kjeldsen K. U. Lever M. A. Steen A. D.. (2013). Predominant archaea in marine sediments degrade detrital proteins. Nature 496, 215–218. doi: 10.1038/nature12033, PMID: 23535597

[ref55] Logares R. Tesson S. V. Canbäck B. Pontarp M. Hedlund K. Rengefors K. (2018). Contrasting prevalence of selection and drift in the community structuring of bacteria and microbial eukaryotes. Environ. Microbiol. 20, 2231–2240. doi: 10.1111/1462-2920.14265, PMID: 29727053

[ref56] Longhurst A. Sathyendranath S. Platt T. Caverhill C. (1995). An estimate of global primary production in the ocean from satellite radiometer data. J. Plankton Res. 17, 1245–1271. doi: 10.1093/plankt/17.6.1245

[ref57] Lu Z. Gan J. Dai M. Liu H. Zhao X. (2018). Joint effects of extrinsic biophysical fluxes and intrinsic hydrodynamics on the formation of hypoxia west off the Pearl River estuary. J. Geophys. Res. Oceans 123, 6241–6259. doi: 10.1029/2018JC014199

[ref58] Lu M. Wang X. Li H. Jiao J. Luo X. Luo M.. (2022). Microbial community assembly and co-occurrence relationship in sediments of the river-dominated estuary and the adjacent shelf in the wet season. Environ. Pollut. 308:119572. doi: 10.1016/j.envpol.2022.119572, PMID: 35661808

[ref59] Ma L. Tan S. Liu H. Kao S.-J. Dai M. Yang J.-Y. T. (2021). Distribution and activity of Ammonia-oxidizers on the size-fractionated particles in the Pearl River estuary. Front. Mar. Sci. 8:685955. doi: 10.3389/fmars.2021.685955

[ref60] McLusky D.S. Elliott M. (2004). The estuarine ecosystem: ecology, threats and management. Oxford: OUP Oxford.

[ref61] Megonigal J. Hines M. Visscher P. (2004). Anaerobic metabolism: linkages to trace gases. Biogeochemistry 8, 317–424. doi: 10.1016/B0-08-043751-6/08132-9

[ref62] Mo Y. Peng F. Gao X. Xiao P. Logares R. Jeppesen E.. (2021). Low shifts in salinity determined assembly processes and network stability of microeukaryotic plankton communities in a subtropical urban reservoir. Microbiome 9, 128–117. doi: 10.1186/s40168-021-01079-w, PMID: 34082826 PMC8176698

[ref63] Morriën E. Hannula S. E. Snoek L. B. Helmsing N. R. Zweers H. De Hollander M.. (2017). Soil networks become more connected and take up more carbon as nature restoration progresses. Nat. Commun. 8, 1–10. doi: 10.1038/ncomms14349, PMID: 28176768 PMC5309817

[ref64] Mougi A. Kondoh M. (2012). Diversity of interaction types and ecological community stability. Science 337, 349–351. doi: 10.1126/science.1220529 22822151

[ref65] Oksanen J. Blanchet F.G. Friendly M. Kindt R. Legendre P. McGlinn D.. (2020). The Vegan Package. Community Ecology Package Version 2.5-7.

[ref66] Oksanen J. Blanchet F. G. Kindt R. Legendre P. Minchin P. R. O’hara R.. (2013). Package ‘vegan’. Commun. Ecol. Pack. 2, 1–295.

[ref67] Olesen J. M. Bascompte J. Dupont Y. L. Jordano P. (2007). The modularity of pollination networks. Proc. Natl. Acad. Sci. U. S. A. 104, 19891–19896. doi: 10.1073/pnas.0706375104, PMID: 18056808 PMC2148393

[ref68] Oren A. (2011). Thermodynamic limits to microbial life at high salt concentrations. Environ. Microbiol. 13, 1908–1923. doi: 10.1111/j.1462-2920.2010.02365.x, PMID: 21054738

[ref69] Orphan V. J. House C. H. Hinrichs K.-U. McKeegan K. D. DeLong E. F. (2002). Multiple archaeal groups mediate methane oxidation in anoxic cold seep sediments. Proc. Natl. Acad. Sci. 99, 7663–7668. doi: 10.1073/pnas.072210299, PMID: 12032340 PMC124316

[ref70] Östman Ö. Drakare S. Kritzberg E. Langenheder S. Logue J. Lindström E. (2010). Regional invariance among microbial communities. Ecol. Lett. 13, 118–127. doi: 10.1111/j.1461-0248.2009.01413.x, PMID: 19968693

[ref71] Pandit S. N. Kolasa J. Cottenie K. (2009). Contrasts between habitat generalists and specialists: an empirical extension to the basic metacommunity framework. Ecology 90, 2253–2262. doi: 10.1890/08-0851.1, PMID: 19739387

[ref72] Parks D. H. Tyson G. W. Hugenholtz P. Beiko R. G. (2014). STAMP: statistical analysis of taxonomic and functional profiles. Bioinformatics 30, 3123–3124. doi: 10.1093/bioinformatics/btu494, PMID: 25061070 PMC4609014

[ref73] Peng G.-S. Wu J. (2016). Optimal network topology for structural robustness based on natural connectivity. Phys. Stat. Mech. Appl. 443, 212–220. doi: 10.1016/j.physa.2015.09.023

[ref74] Quast C. Pruesse E. Yilmaz P. Gerken J. Schweer T. Yarza P.. (2013). The SILVA ribosomal RNA gene database project: improved data processing and web-based tools. Nucleic Acids Res. 41, D590–D596. doi: 10.1093/nar/gks1219, PMID: 23193283 PMC3531112

[ref75] Röttjers L. Faust K. (2018). From hairballs to hypotheses–biological insights from microbial networks. FEMS Microbiol. Rev. 42, 761–780. doi: 10.1093/femsre/fuy030, PMID: 30085090 PMC6199531

[ref76] Schleper C. Jurgens G. Jonuscheit M. (2005). Genomic studies of uncultivated archaea. Nat. Rev. Microbiol. 3, 479–488. doi: 10.1038/nrmicro1159 15931166

[ref77] Schloss P. D. Westcott S. L. Ryabin T. Hall J. R. Hartmann M. Hollister E. B.. (2009). Introducing mothur: open-source, platform-independent, community-supported software for describing and comparing microbial communities. Appl. Environ. Microbiol. 75, 7537–7541. doi: 10.1128/AEM.01541-09, PMID: 19801464 PMC2786419

[ref78] Shi Y. Fan K. Li Y. Yang T. He J. S. Chu H. (2019). Archaea enhance the robustness of microbial co-occurrence networks in Tibetan plateau soils. Soil Sci. Soc. Am. J. 83, 1093–1099. doi: 10.2136/sssaj2018.11.0426

[ref79] Shi Y. Li Y. Xiang X. Sun R. Yang T. He D.. (2018). Spatial scale affects the relative role of stochasticity versus determinism in soil bacterial communities in wheat fields across the North China plain. Microbiome 6, 27–12. doi: 10.1186/s40168-018-0409-4, PMID: 29402331 PMC5799910

[ref80] Sloan W. Lunn M. Woodcock S. Head I. Nee S. Curtis T. (2006). Quantifying the roles of immigration and chance in shaping prokaryote community structure. Environ. Microbiol. 8, 732–740. doi: 10.1111/j.1462-2920.2005.00956.x, PMID: 16584484

[ref81] Stegen J. C. Lin X. Fredrickson J. K. Konopka A. E. (2015). Estimating and mapping ecological processes influencing microbial community assembly. Front. Microbiol. 6:370. doi: 10.3389/fmicb.2015.00370, PMID: 25983725 PMC4416444

[ref82] Stone L. Roberts A. (1990). The checkerboard score and species distributions. Oecologia 85, 74–79. doi: 10.1007/BF00317345, PMID: 28310957

[ref83] Swenson N. (2014). Functional and Phylogenetic Ecology in R. Springer Science & Business Media.

[ref84] R Core Team R. (2013). R: A language and environment for statistical computing.

[ref85] Vellend M. Srivastava D. S. Anderson K. M. Brown C. D. Jankowski J. E. Kleynhans E. J.. (2014). Assessing the relative importance of neutral stochasticity in ecological communities. Oikos 123, 1420–1430. doi: 10.1111/oik.01493

[ref86] Vieira R. P. Clementino M. M. Cardoso A. M. Oliveira D. N. Albano R. M. Gonzalez A. M.. (2007). Archaeal communities in a tropical estuarine ecosystem: Guanabara Bay Brazil. Microb. Ecol. 54, 460–468. doi: 10.1007/s00248-007-9261-y, PMID: 17593423

[ref87] Wang P. Zhang T. Chen S. Li X. Lai D. Gao S.. (2020). Niche specificity and potential terrestrial organic carbon utilization of benthic Bathyarchaeota in a eutrophic subtropic estuarine system. Chem. Geol. 556:119839. doi: 10.1016/j.chemgeo.2020.119839

[ref88] Webster G. O'Sullivan L. A. Meng Y. Williams A. S. Sass A. M. Watkins A. J.. (2015). Archaeal community diversity and abundance changes along a natural salinity gradient in estuarine sediments. FEMS Microbiol. Ecol. 91, 1–18. doi: 10.1093/femsec/fiu025, PMID: 25764553 PMC4399439

[ref89] Wei G. Li M. Li F. Li H. Gao Z. (2016). Distinct distribution patterns of prokaryotes between sediment and water in the Yellow River estuary. Appl. Microbiol. Biotechnol. 100, 9683–9697. doi: 10.1007/s00253-016-7802-3, PMID: 27557722

[ref90] Wen X. Yang S. Horn F. Winkel M. Wagner D. Liebner S. (2017). Global biogeographic analysis of methanogenic Archaea identifies community-shaping environmental factors of natural environments. Front. Microbiol. 8:1339. doi: 10.3389/fmicb.2017.01339, PMID: 28769904 PMC5513909

[ref91] Wu W. Lu H.-P. Sastri A. Yeh Y.-C. Gong G.-C. Chou W.-C.. (2018). Contrasting the relative importance of species sorting and dispersal limitation in shaping marine bacterial versus protist communities. ISME J. 12, 485–494. doi: 10.1038/ismej.2017.183, PMID: 29125596 PMC5776463

[ref92] Xie W. Luo H. Murugapiran S. K. Dodsworth J. A. Chen S. Sun Y.. (2018). Localized high abundance of marine group II archaea in the subtropical Pearl River estuary: implications for their niche adaptation. Environ. Microbiol. 20, 734–754. doi: 10.1111/1462-2920.14004, PMID: 29235710

[ref93] Xie W. Zhang C. L. Zhou X. D. Wang P. (2014). Salinity-dominated change in community structure and ecological function of Archaea from the lower Pearl River to coastal South China Sea. Appl. Microbiol. Biotechnol. 98, 7971–7982. doi: 10.1007/s00253-014-5838-9, PMID: 24880629

[ref94] Yin K. D. (2002). Monsoonal influence on seasonal variations in nutrients and phytoplankton biomass in coastal waters of Hong Kong in the vicinity of the Pearl River estuary. Mar. Ecol. Prog. Ser. 245, 111–122. doi: 10.3354/meps245111

[ref95] Zhang C.-J. Pan J. Duan C.-H. Wang Y.-M. Liu Y. Sun J.. (2019). Prokaryotic diversity in mangrove sediments across southeastern China fundamentally differs from that in other biomes. Msystems 4, e00442–e00419. doi: 10.1128/mSystems.00442-19, PMID: 31506265 PMC6739103

[ref96] Zhang Z.-F. Pan J. Pan Y.-P. Li M. (2021). Biogeography, assembly patterns, driving factors, and interactions of archaeal community in mangrove sediments. Msystems 6, e0138120–e0101320. doi: 10.1128/mSystems.01381-20, PMID: 34128692 PMC8269266

[ref97] Zhang W. Pan Y. Yang J. Chen H. Holohan B. Vaudrey J.. (2018). The diversity and biogeography of abundant and rare intertidal marine microeukaryotes explained by environment and dispersal limitation. Environ. Microbiol. 20, 462–476. doi: 10.1111/1462-2920.13916, PMID: 28881067

[ref98] Zhou Z. Liu Y. Lloyd K. G. Pan J. Yang Y. Gu J.-D.. (2019). Genomic and transcriptomic insights into the ecology and metabolism of benthic archaeal cosmopolitan, Thermoprofundales (MBG-D archaea). ISME J. 13, 885–901. doi: 10.1038/s41396-018-0321-8, PMID: 30514872 PMC6461988

[ref99] Zhou Z. Meng H. Liu Y. Gu J.-D. Li M. (2017). Stratified bacterial and archaeal community in mangrove and intertidal wetland mudflats revealed by high throughput 16S rRNA gene sequencing. Front. Microbiol. 8:2148. doi: 10.3389/fmicb.2017.02148, PMID: 29163432 PMC5673634

[ref100] Zhou J. Ning D. (2017). Stochastic community assembly: does it matter in microbial ecology? Microbiol. Mol. Biol. Rev. 81, e00002–e00017. doi: 10.1128/MMBR.00002-17, PMID: 29021219 PMC5706748

[ref101] Zhou H.-Y. Peng X.-T. Pan J.-M. (2004). Distribution, source and enrichment of some chemical elements in sediments of the Pearl River estuary China. Cont. Shelf Res. 24, 1857–1875. doi: 10.1016/j.csr.2004.06.012

[ref102] Zou D. Liu H. Li M. (2020a). Community, distribution, and ecological roles of estuarine Archaea. Front. Microbiol. 11:2060. doi: 10.3389/fmicb.2020.02060, PMID: 32983044 PMC7484942

[ref103] Zou D. Pan J. Liu Z. Zhang C. Liu H. Li M. (2020b). The distribution of Bathyarchaeota in surface sediments of the Pearl River estuary along salinity gradient. Front. Microbiol. 11:285. doi: 10.3389/fmicb.2020.00285, PMID: 32174899 PMC7056671

