# Supplementary material for: Deterministic processes dominate archaeal community assembly from the Pearl River to the northern South China Sea
Source: Front Microbiol. 2023 Jun 22;14:1185436. doi: 10.3389/fmicb.2023.1185436 (PMC10324572; doi:10.3389/fmicb.2023.1185436)
Supplement: Supplementary file 1 [file Data_Sheet_1.PDF]

Supplementary Material

Deterministic processes dominate archaeal community assembly from the Pearl River to the northern South China Sea

Xizheng Lin[1,2], Chuanlun Zhang[4,5,6], Wei Xie[1,2,3*]

*** Correspondence:** Wei Xie: xiewei9@mail.sysu.edu.cn

List of figures

Figure S1. Rarefaction curves of the Shannon index using (A) 16S rRNA sequences and (B) *amoA* sequences at different sampling depth. The dotted lines represent the subsampled depth.

Figure S2. Bray-Curtis dissimilarity-based dendrogram at the operational taxonomic unit (OTU) level of 16S rRNA sequences illustrating groups (A, low-salinity-water and high-salinity-water; B, low-salinity-sediment and high-salinity-sediment) in samples.

Figure S3. Designated OTUs based on the *amoA* gene were mapped to a specific evolutionary tree (Alves et al., 2018) to determine their classification. The size of red nodes represents the number of OTUs mapped to a specific phylogenetic clade.

Figure S4. Community composition of AOA based on *amoA* gene.

Figure S5. Principal component analysis (PCA) of total archaeal community based on 16s rRNA gene (A) and AOA composition based on *amoA* gene (B) in OTU levels.

Figure S6. Results of difference test of alpha diversity indexes between groups based on (A) 16S rRNA genes and (B) *amoA* genes. Kruskal-Wallis test and Dunn's test were applied for the significance test. The single asterisk (*), double asterisk (**), and three asterisk (***) indicated the significant difference at $P < 0.05$, $P < 0.01$, and $P < 0.001$ levels, respectively.

Figure S7. The bubble chart of relative abundance of OTUs occurred in more than 70% of (A) water and (B) sediment samples. The size of nodes represent the relative abundance of OTUs.

Figure S8. Fit of the neutral community model (NCM) of community assembly for all-water and all-sediment groups with the same number of the high-salinity and low-salinity samples.

Figure S9. Co-occurrence networks of the archaeal community based on pairwise Spearman's correlations between OTUs. Each shown connection has a correlation coefficient $|r| > 0.6$ and a P value < 0.05. The red edges represent significant positive correlations, and the blue edges represent significant negative correlations. The size of each node is proportional to the number of connections. The upper panel shows the network of water samples with OTUs colored by taxonomy (A) and modularity (B); the lower panel shows the network of sediment samples with OTUs colored by

taxonomy (D) and modularity (E). Relative abundance of archaeal OTUs from major modules in water (C) and sediment (F).

Figure S10. Network stability of archaeal communities in different groups.

Figure S11. Results of difference test of multiple environmental variables between the high-salinity-water and low-salinity-water groups. Mann-Whitney U-test was applied for the significance test. The single asterisk (*) and double asterisk (**) indicated the significant difference at $P < 0.05$ and $P < 0.01$ levels, respectively.

Figure S12. Difference in environmental heterogeneity in the high-salinity-water and low-salinity-water groups. Mann-Whitney U-test was applied for the significance test. The single asterisk (*) indicated the significant difference at $P < 0.05$.

Figure S13. Fit of the neutral community model (NCM) of community assembly for high-salinity environments, which had the same sample number to low-salinity environments.

List of tables

Table S1. Physicochemical parameters and alpha diversity indexes of samples.

Table S2. Designated AOA OTUs based on the *amoA* gene were mapped to a specific evolutionary tree (Alves et al., 2018) to determine their phylogenetic clades.

Table S3. The analysis of similarities (ANOSIM) for total archaeal community composition in OTU level among groups.

Table S4. The analysis of similarities (ANOSIM) for AOA composition in OTU level among groups.

Table S5. Topological properties of the empirical species co-occurrence networks of archaeal communities and their associated random networks.

Table S6. Number of degrees of different archaeal groups in eight major modules in planktonic archaeal network from the PR to the NSCS.

Table S7. Number of degrees of different archaeal groups in eight major modules in benthic archaeal network from the PR to the NSCS.

Table S8. Mean habitat niche breadth for all taxa among the high-salinity-water, low-salinity-water, high-salinity-sediment, and low-salinity-sediment groups.

Supplemental Material FOR Figures

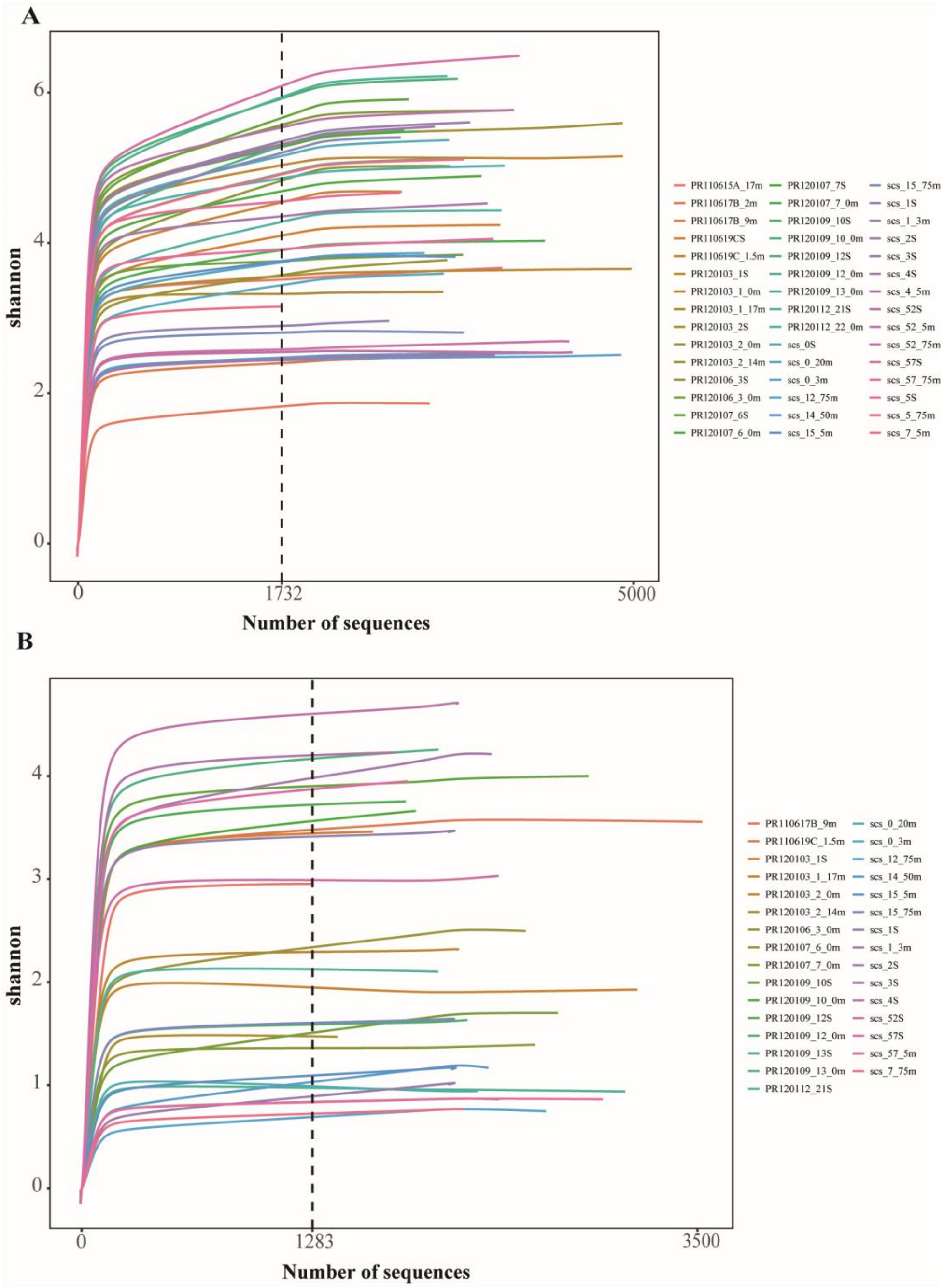

FIG S1 Rarefaction curves of the Shannon index using (A) 16S rRNA sequences and (B) *amoA* sequences at different sampling depth. The dotted lines represent the subsampled depth.

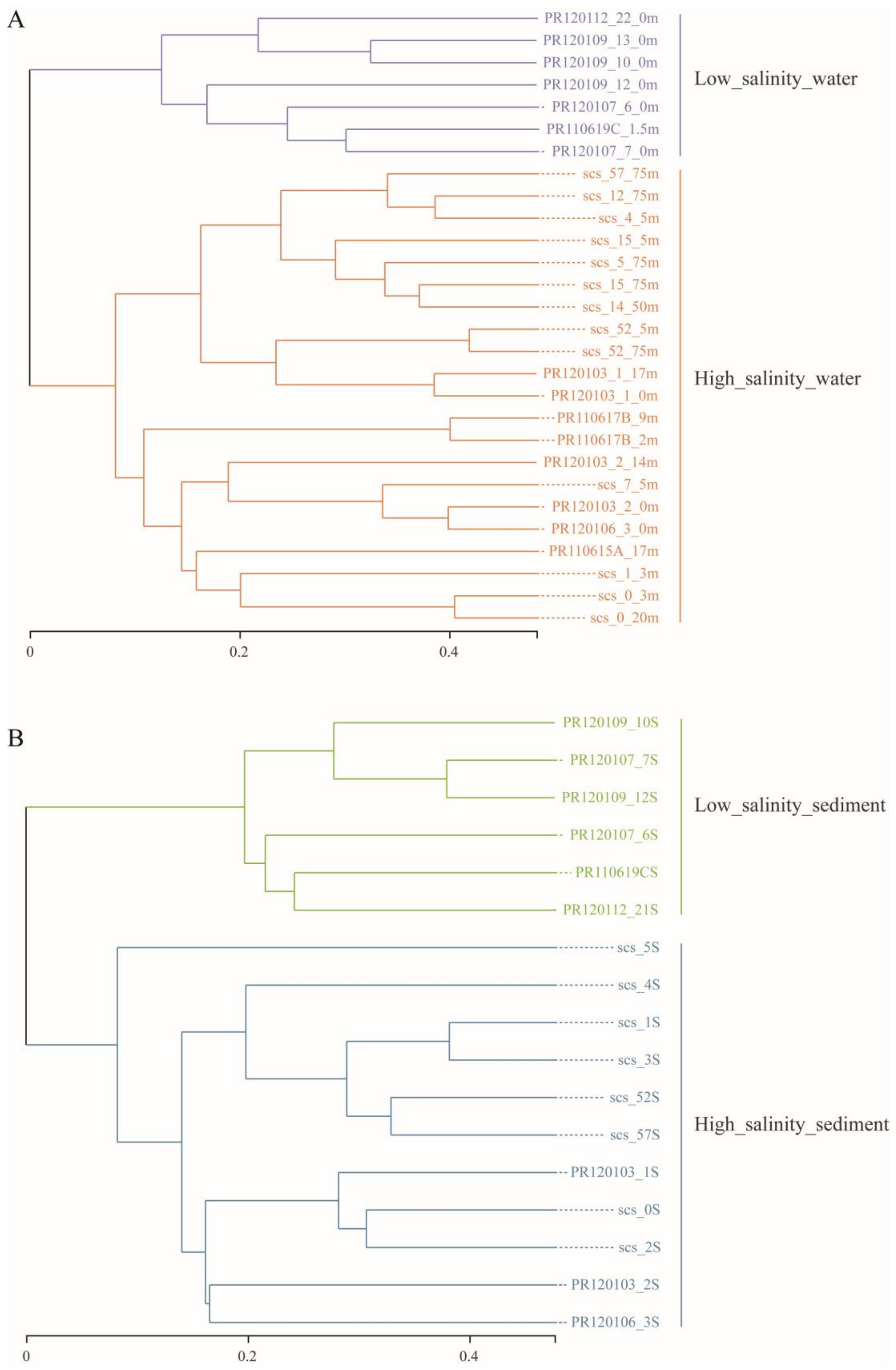

FIG S2 Bray-Curtis dissimilarity-based dendrogram at the operational taxonomic unit (OTU) level of 16S rRNA sequences illustrating groups (A, low-salinity-water and high-salinity-water; B, low-salinity-sediment and high-salinity-sediment) in samples.

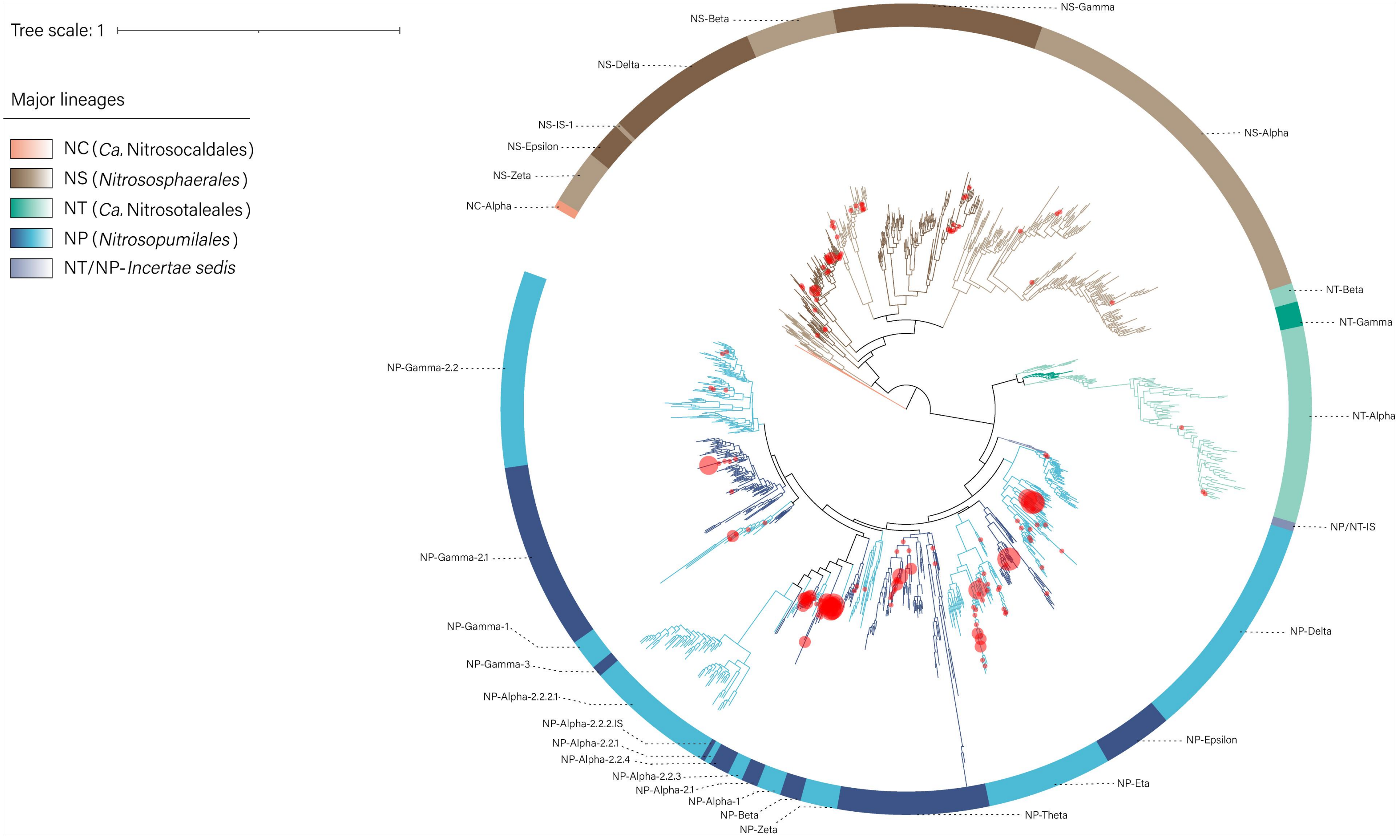

FIG S3 Designated OTUs based on the *amoA* gene were mapped to a specific evolutionary tree (Alves et al., 2018) to determine their classification. The size of red nodes represents the number of OTUs mapped to a specific phylogenetic clade.

frontiers

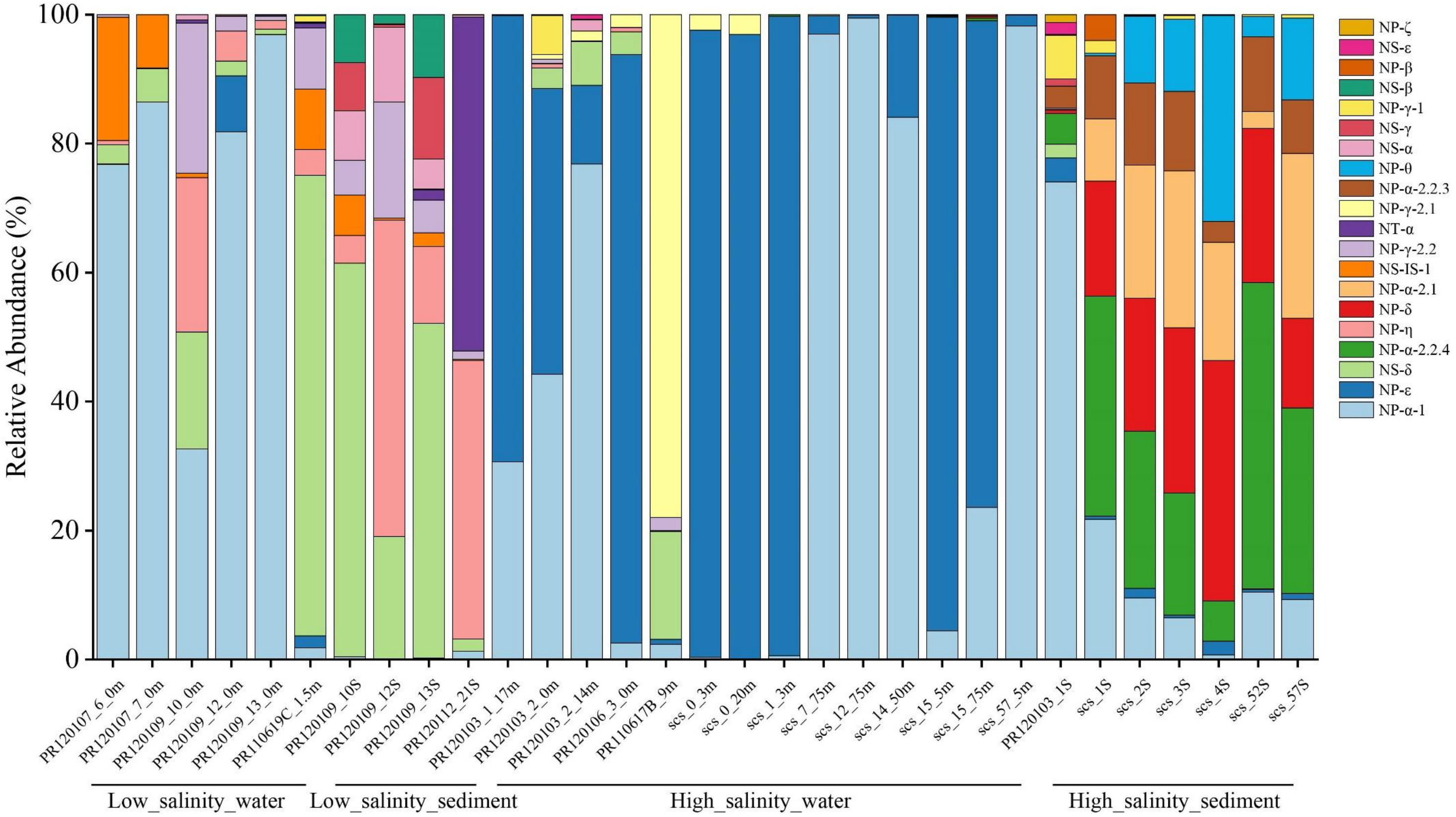

FIG S4 Community composition of AOA based on *amoA* gene.

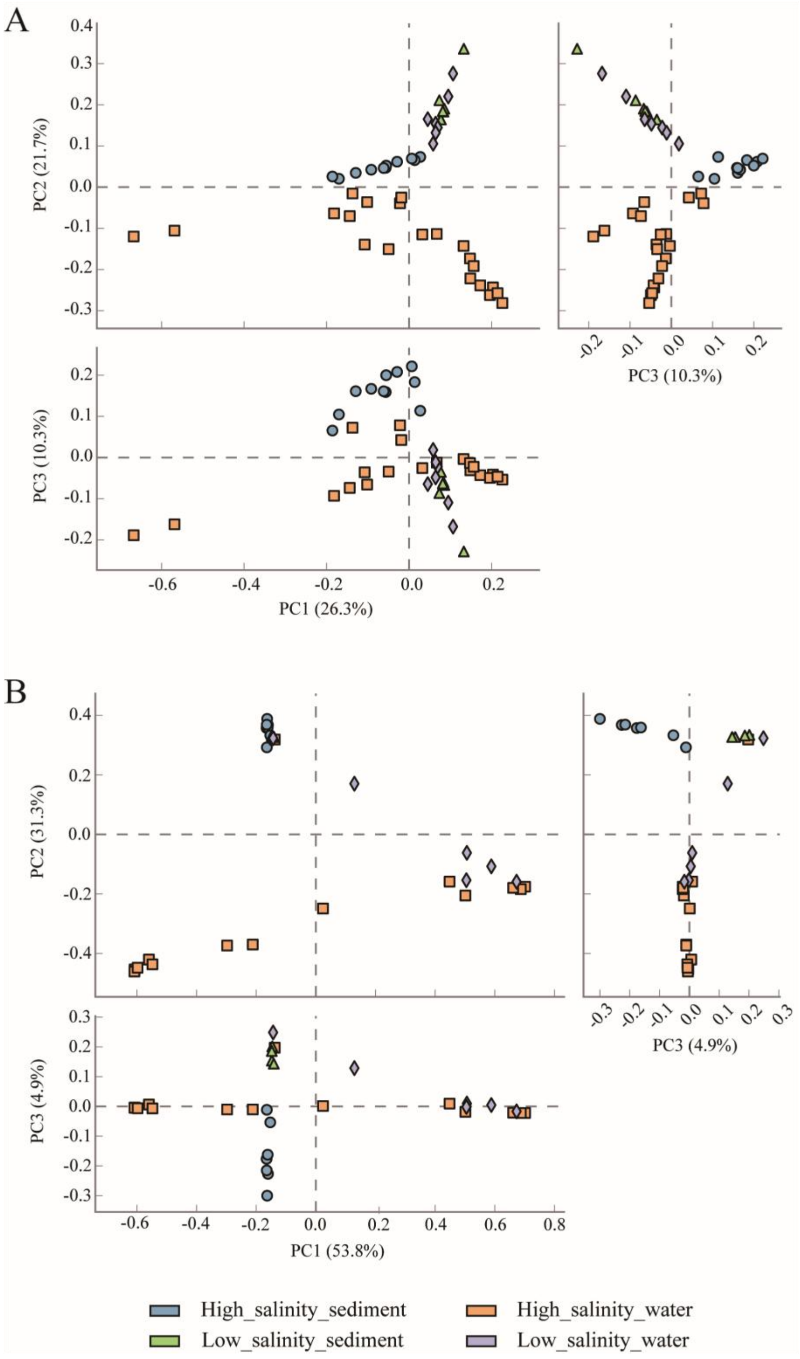

FIG S5 Principal component analysis (PCA) of total archaeal community based on 16s rRNA gene (A) and AOA composition based on *amoA* gene (B) in OTU levels.

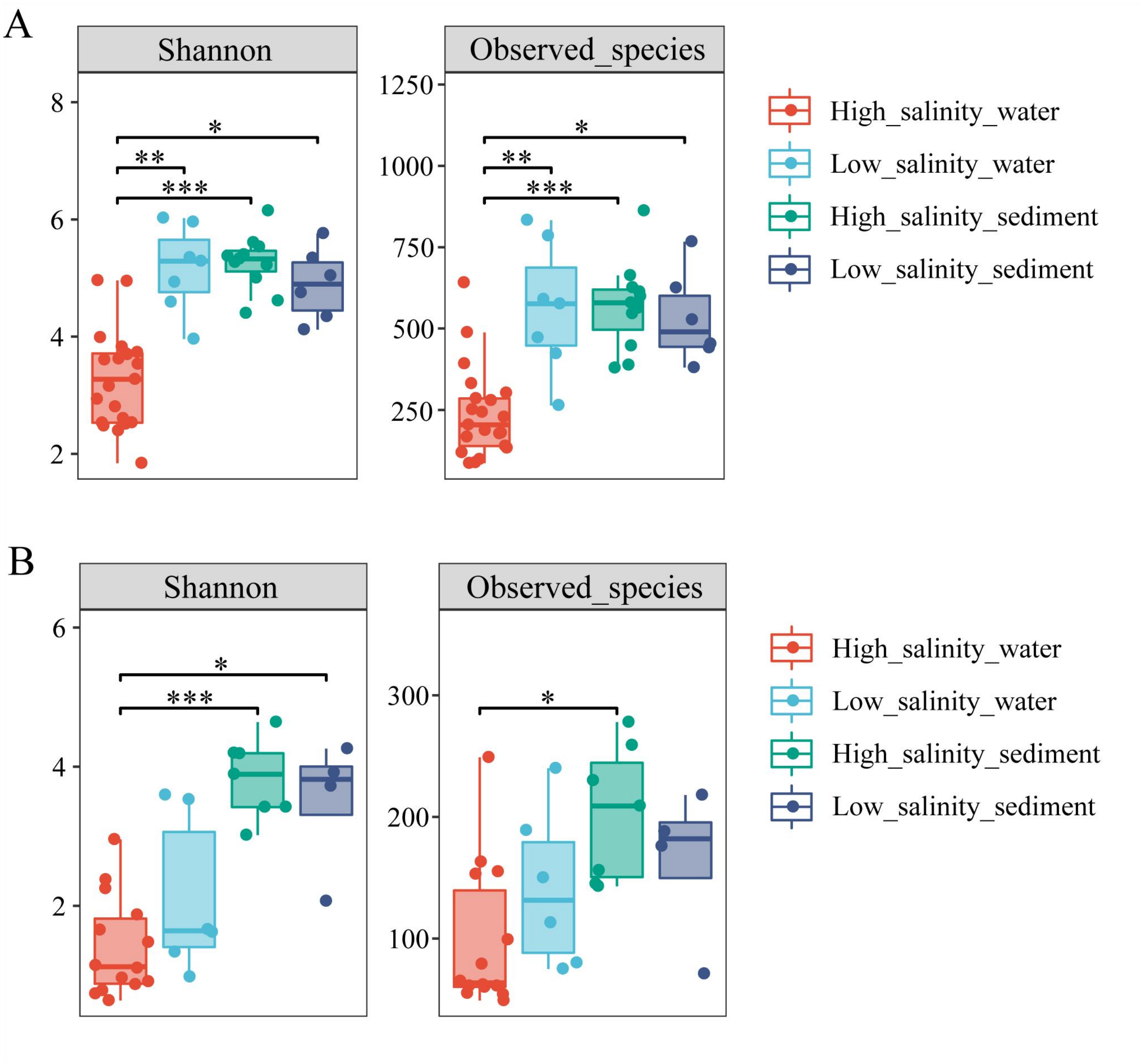

FIG S6 Results of difference test of alpha diversity indexes between groups based on (A) 16S rRNA genes and (B) *amoA* genes. Kruskal-Wallis test and Dunn's test were applied for the significance test. The single asterisk (*), double asterisk (**), and three asterisk (***) indicated the significant difference at $P < 0.05$, $P < 0.01$, and $P < 0.001$ levels, respectively.

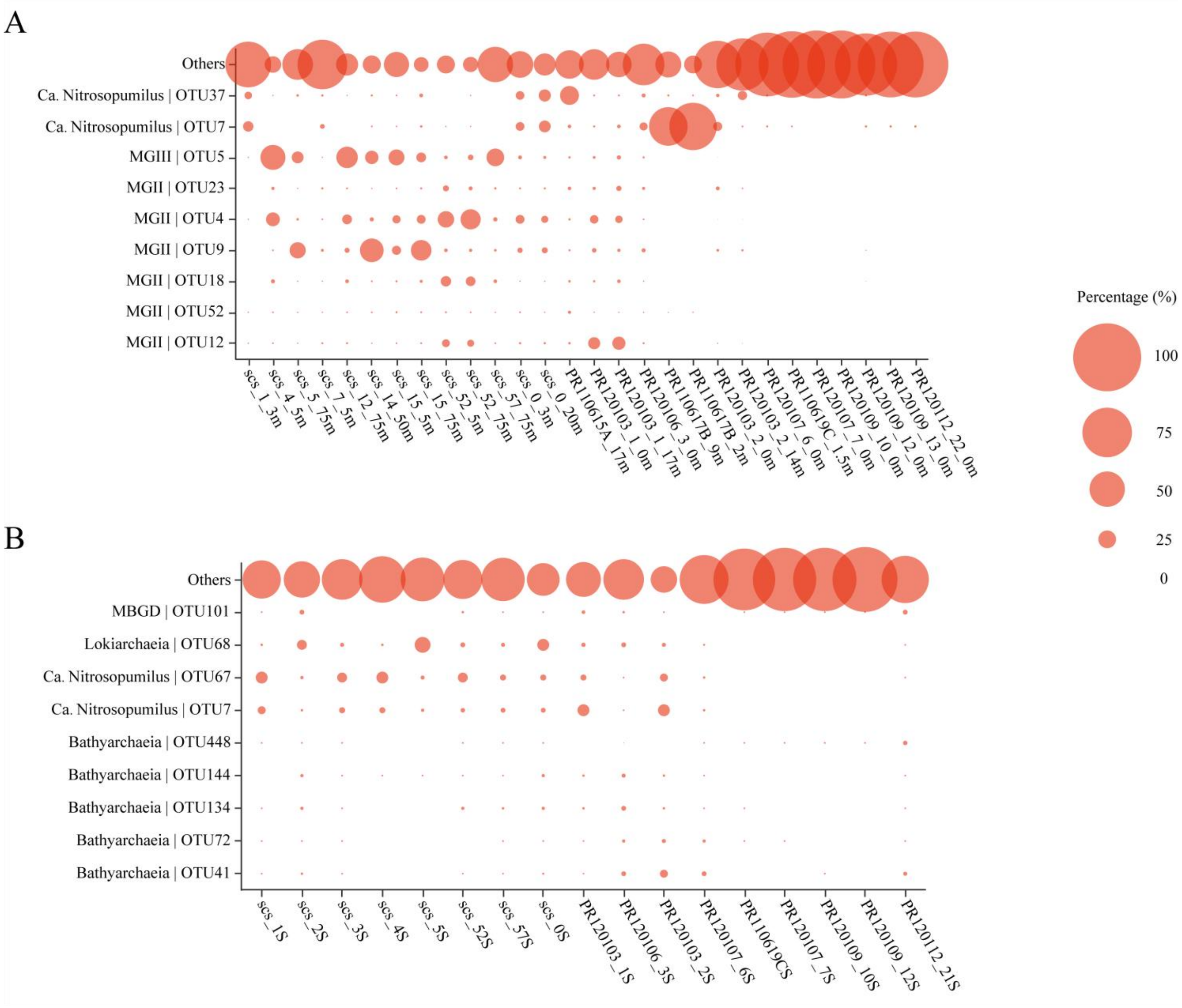

FIG S7 The bubble chart of relative abundance of OTUs occurred in more than 70% of (A) water and (B) sediment samples. The size of nodes represent the relative abundance of OTUs.

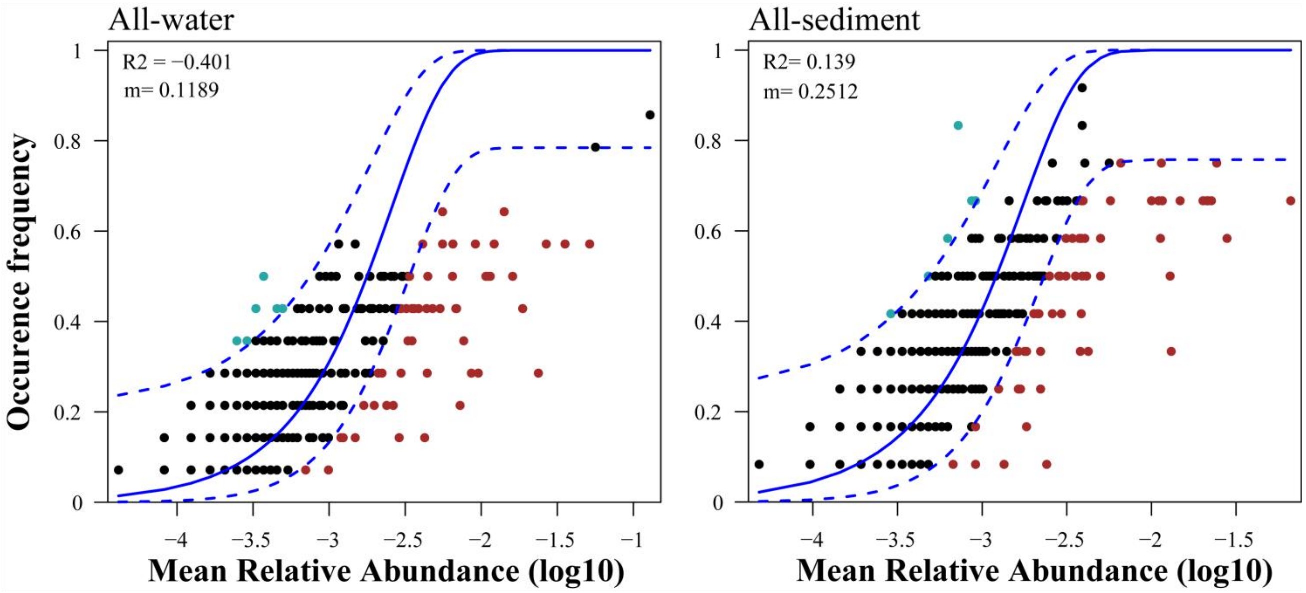

FIG S8 Fit of the neutral community model (NCM) of community assembly for all-water and all-sediment groups with the same number of the high-salinity and low-salinity samples.

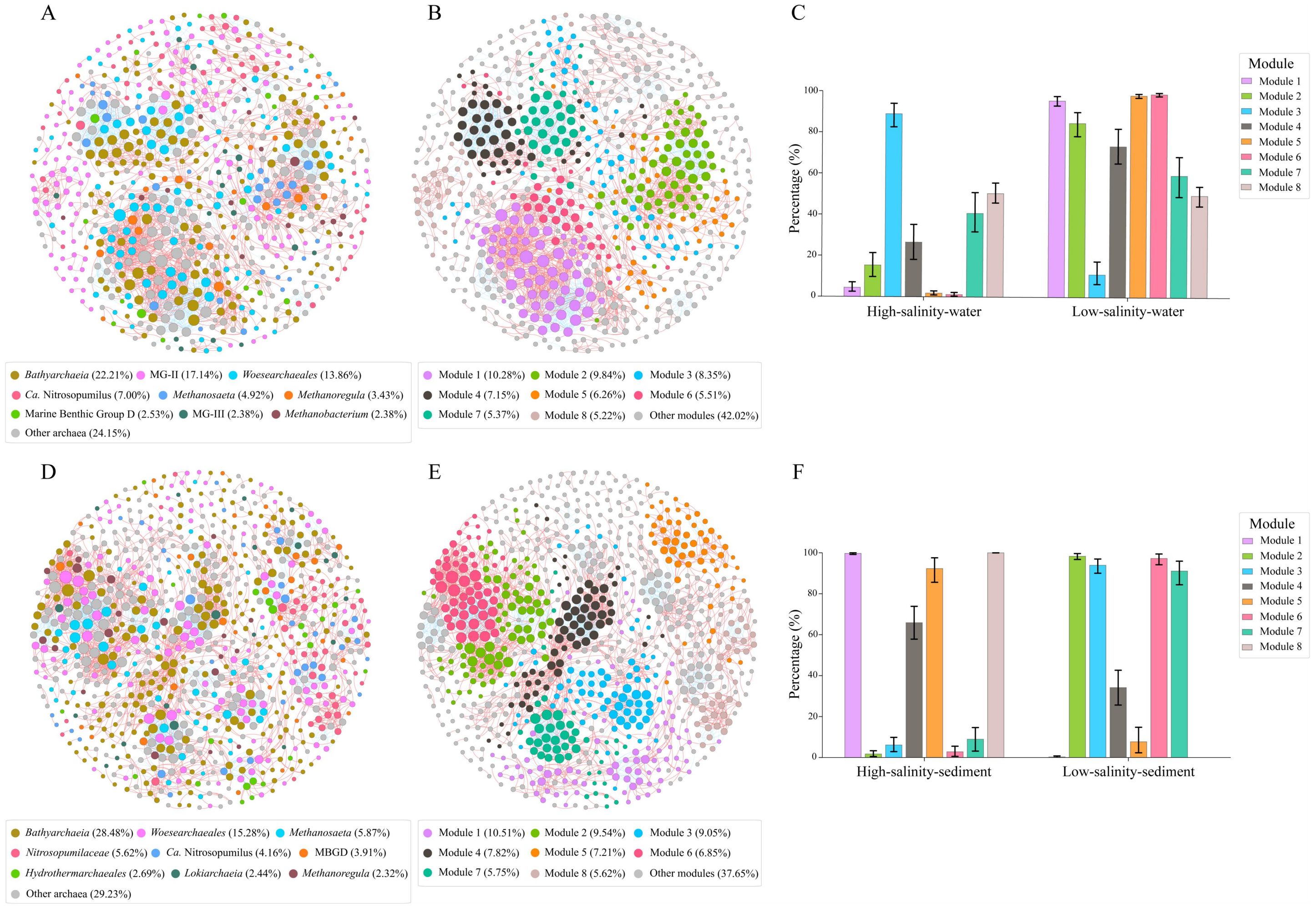

FIG S9 Co-occurrence networks of the archaeal community based on pairwise Spearman's correlations between OTUs. Each shown connection has a correlation coefficient >|0.6| and a P value < 0.05. The size of each node is proportional to the number of connections. The upper panel shows the network of water samples with OTUs colored by taxonomy (A) and modularity (B); the lower panel shows the network of sediment samples with OTUs colored by taxonomy (D) and modularity (E). Relative abundance of archaeal OTUs from major modules in water (C) and sediment (F).

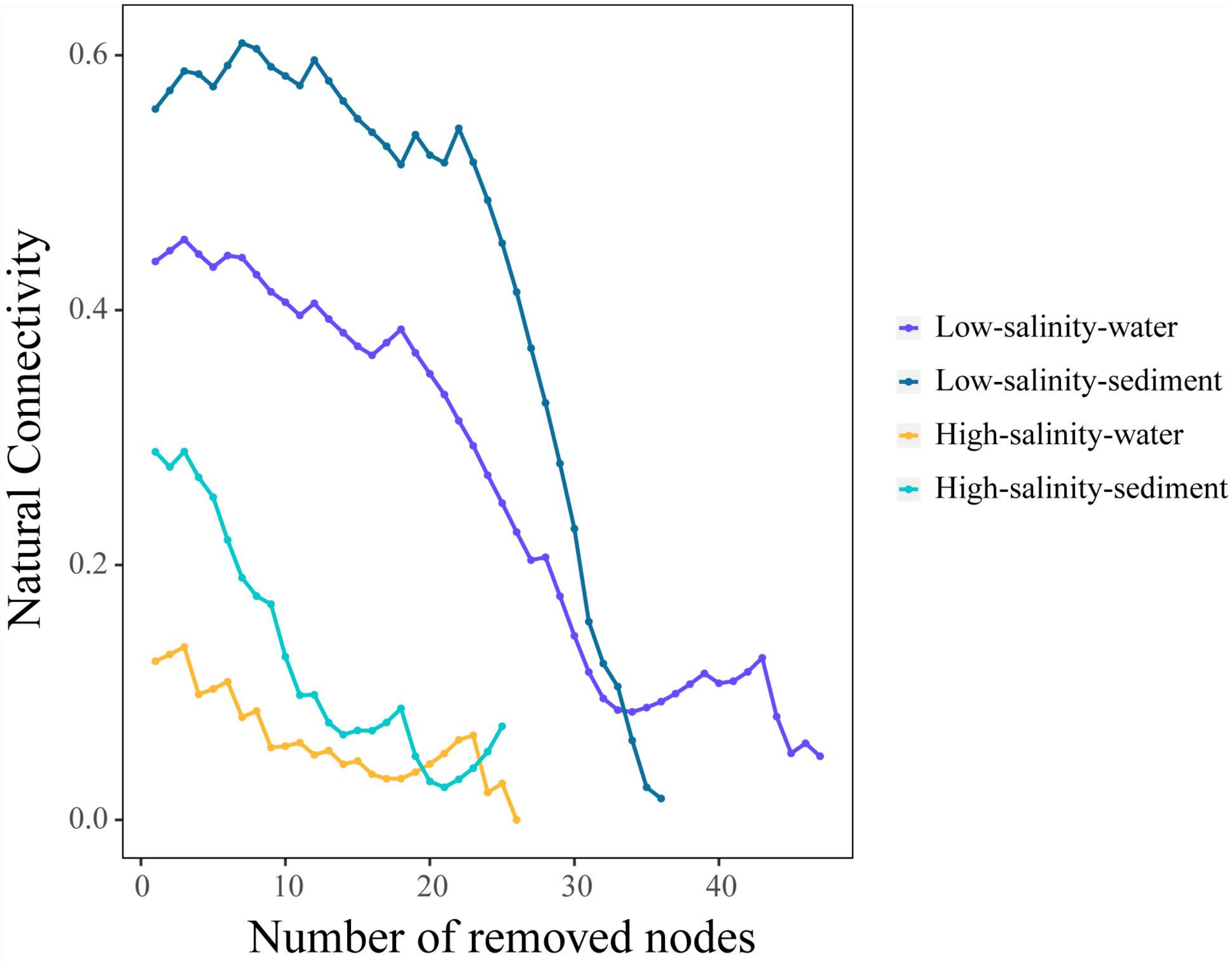

FIG S10 Network stability of archaeal communities in different groups.

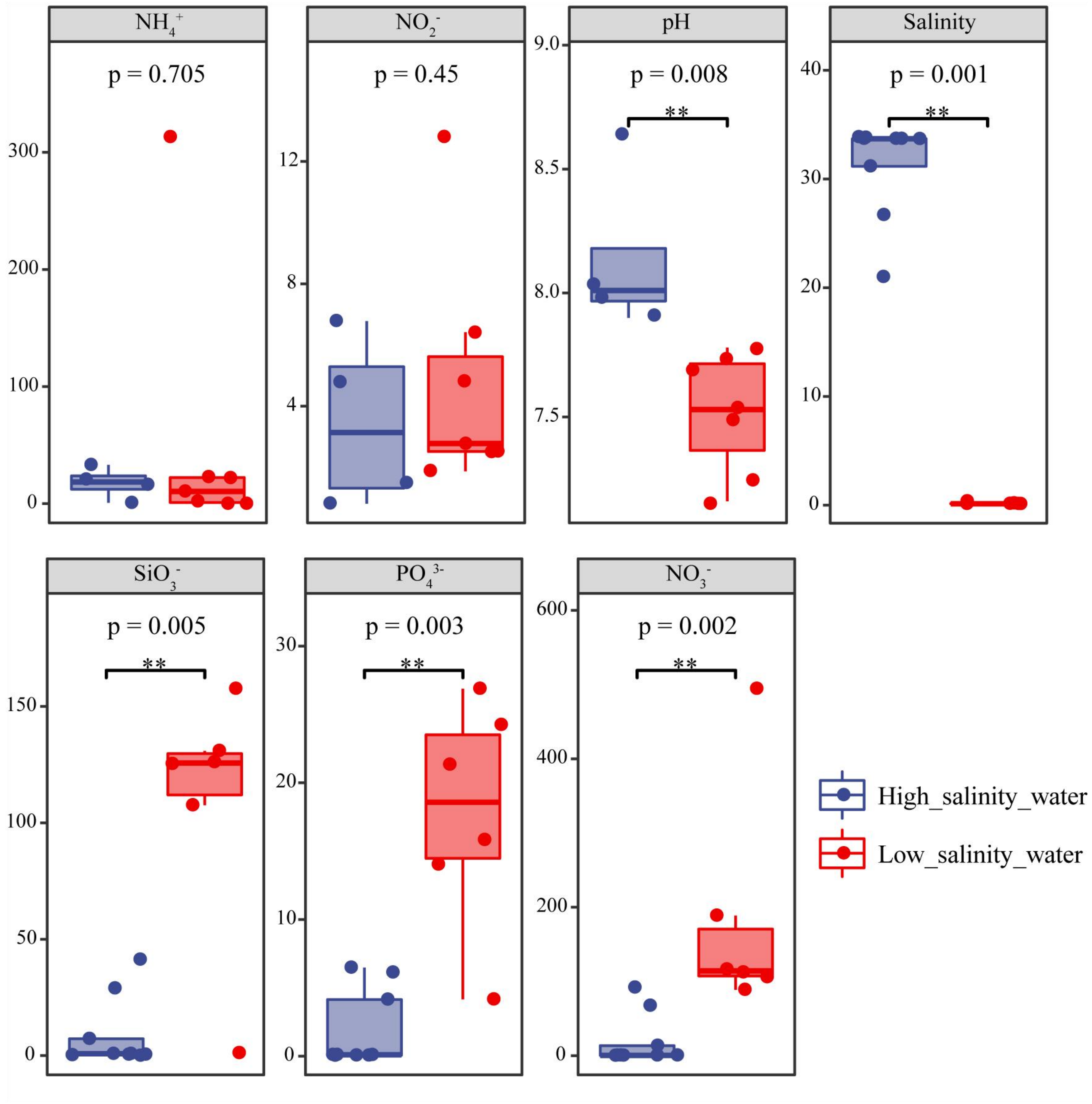

FIG S11 Results of difference test of multiple environmental variables between the high-salinity-water and low-salinity-water groups. Mann-Whitney U-test was applied for the significance test. The single asterisk (*) and double asterisk (**) indicated the significant difference at $P < 0.05$ and $P < 0.01$ levels, respectively.

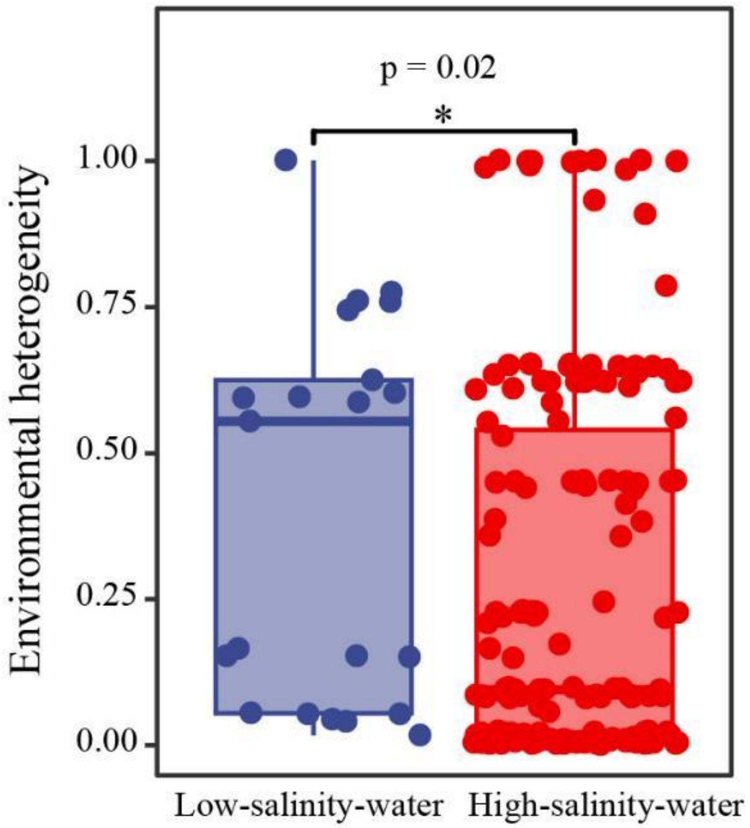

FIG S12 Difference in environmental heterogeneity in the high-salinity-water and low-salinity-water groups. Mann-Whitney U-test was applied for the significance test. The single asterisk (*) indicated the significant difference at $P < 0.05$.

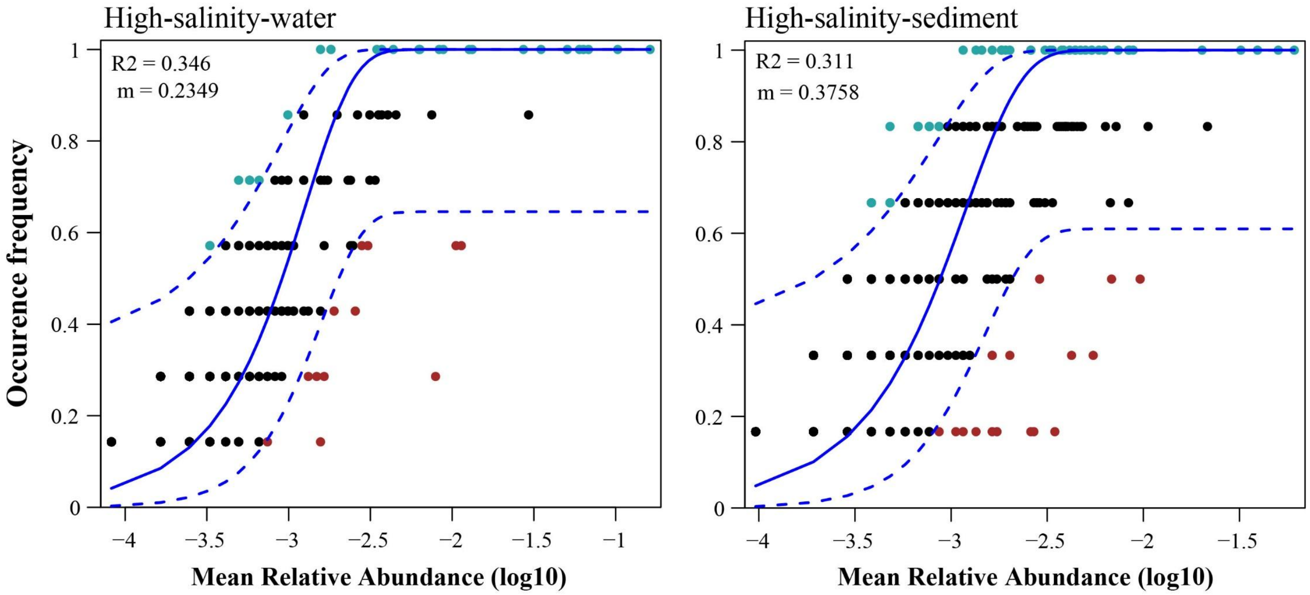

FIG S13 Fit of the neutral community model (NCM) of community assembly for high-salinity environments, which had the same sample number to low-salinity environments.

Supplemental Material FOR Tables

TABLE S1 Physicochemical parameters and alpha diversity indexes of samples.

Sample ID	Date	Longitude (E)	Latitude (N)	NH_4^+ (μM)	NO_3^- (μM)	NO_2^- (μM)	SiO_3^{2-} (μM)	PO_4^{3-} (μM)	pH	Temp (℃)	Salinity (‰)	Shannon	Simpson	Observed_species	Shannon_amoA	Observed_species_amoA	Filter volumn (L)
PR120103_1_0m	2012.1.3	113.697	21.943	0.760	13.550	0.810	7.230	4.144	8.03	17.63	31.17	3.622	0.933	228	n.a	n.a	5.83
PR120103_1_17m	2012.1.3	113.697	21.943	n.a	n.a	n.a	n.a	n.a	n.a	n.a	n.a	3.273	0.922	177	1.871	153	5.83
PR120103_2_0m	2012.1.3	113.717	22.571	20.720	91.940	6.780	41.270	6.481	7.99	14.00	21.00	3.732	0.877	392	2.248	155	3.75
PR120103_2_14m	2012.1.3	113.717	22.571	n.a	n.a	n.a	n.a	n.a	n.a	n.a	28.90	4.946	0.971	488	1.476	79	0.29
PR120106_3_0m	2012.1.6	113.751	22.454	16.210	67.470	4.790	28.910	6.126	7.90	13.18	26.70	3.695	0.900	331	2.377	249	1.00
PR120107_6_0m	2012.1.7	113.571	22.874	n.a	n.a	n.a	n.a	n.a	7.30	16.12	14.58	5.349	0.982	590	1.336	75	2.08
PR120107_7_0m	2012.1.7	113.160	23.055	10.480	494.400	12.800	1.130	21.333	7.16	14.97	0.36	3.958	0.942	264	1.661	150	0.42
PR120109_10_0m	2012.1.9	112.963	23.090	2.000	112.320	2.500	126.110	15.823	7.70	13.56	0.12	5.956	0.993	785	3.594	240	1.00
PR120109_12_0m	2012.1.9	112.825	23.161	0.000	116.340	1.870	107.580	24.241	7.53	14.41	0.13	5.289	0.978	576	1.621	113	0.92
PR120109_13_0m	2012.1.9	112.807	23.219	0.000	105.860	2.530	130.940	14.022	7.48	13.43	0.12	6.023	0.992	833	0.979	80	0.79
PR120112_21_0m	2012.1.12	113.173	23.701	22.780	112.680	2.780	124.140	25.326	7.78	12.54	0.11	n.a	n.a	n.a	n.a	n.a	2.56
PR120112_22_0m	2012.1.12	113.240	23.791	313.080	88.820	4.820	157.640	26.885	7.73	12.43	0.11	4.930	0.979	423	n.a	n.a	3.48
PR110615A_1.5m	2011.6.15	113.704	21.952	33.137	11.775	1.485	0.800	0.559	8.63	29.57	26.54	n.a	n.a	n.a	n.a	n.a	12.92
PR110615A_17m	2011.6.15	113.704	21.952	27.052	36.976	1.098	5.342	0.016	7.92	25.41	33.67	3.605	0.905	251	n.a	n.a	5.42

PR110617B_2m	2011.6.17	113.751	22.454	8.090	149.223	7.122	65.031	1.708	8.03	28.73	21.08	1.840	0.510	187	n.a	n.a	2.46
PR110617B_9m	2011.6.17	113.751	22.454	4.200	93.180	5.694	50.140	1.169	7.89	27.62	22.99	2.396	0.668	204	2.954	163	2.29
PR110619C_1.5m	2011.6.19	113.571	22.877	21.918	188.748	6.415	125.359	4.155	7.25	29.67	0.17	4.590	0.937	472	3.528	189	0.63
scs_0_3m	2012.10	113.708	21.932	n.a	0.496	n.a	0.800	0.099	n.a	26.83	33.71	3.827	0.938	279	0.870	61	4.17
scs_0_20m	2012.10	113.708	21.932	n.a	0.344	n.a	1.488	0.046	n.a	26.75	33.82	3.531	0.916	243	0.912	49	4.17
scs_1_3m	2012.10	113.700	21.500	n.a	0.496	n.a	0.800	0.099	n.a	26.83	33.71	2.935	0.873	179	0.962	61	4.17
scs_4_5m	2012.10	114.600	20.000	n.a	0.392	n.a	0.220	0.060	n.a	27.29	33.81	2.532	0.810	133	n.a	n.a	29.17
scs_5_75m	2012.10	114.896	19.500	n.a	1.528	n.a	3.078	0.204	n.a	22.94	34.35	3.153	0.896	167	n.a	n.a	29.17
scs_7_5m	2012.10	115.501	18.501	n.a	0.300	n.a	0.459	0.093	n.a	27.88	33.69	4.959	0.955	641	n.a	n.a	37.50
scs_7_75m	2012.10	115.501	18.501	n.a	1.864	n.a	3.509	0.289	n.a	22.88	34.36	n.a	n.a	n.a	0.737	60	38.75
scs_12_75m	2012.10	115.020	20.784	n.a	1.460	n.a	1.497	0.116	n.a	23.73	34.35	2.529	0.851	88	0.639	55	4.17
scs_14_50m	2012.10	114.615	21.349	n.a	0.589	n.a	0.513	0.018	n.a	26.32	34.00	2.477	0.824	98	1.105	62	4.17
scs_15_5m	2012.10	114.417	21.624	n.a	0.423	n.a	0.600	0.054	n.a	27.02	33.86	3.714	0.910	285	1.144	65	4.17
scs_15_75m	2012.10	114.417	21.624	n.a	n.a	n.a	n.a	n.a	n.a	n.a	n.a	2.804	0.863	139	1.653	99	4.17
scs_52_5m	2012.10	112.164	21.047	n.a	0.139	n.a	0.459	0.040	n.a	26.83	33.71	2.510	0.870	86	n.a	n.a	2.50
scs_52_75m	2012.10	112.164	21.047	n.a	n.a	n.a	n.a	n.a	n.a	n.a	n.a	2.605	0.859	119	n.a	n.a	0.63
scs_57_5m	2012.10	112.698	20.377	n.a	0.601	n.a	0.858	0.046	n.a	27.15	33.61	n.a	n.a	n.a	0.782	54	6.25
scs_57_75m	2012.10	112.698	20.377	n.a	n.a	n.a	n.a	n.a	n.a	n.a	n.a	3.985	0.918	302	n.a	n.a	6.25
PR120103_1S	2012.1.3	113.697	21.943	87.100	n.a	1.000	140.100	5.700	8.00	17.60	31.20	5.001	0.976	447	3.420	145	n.a

PR120103_2S	2012.1.3	113.717	22.571	121.200	n.a	0.600	75.000	1.100	8.00	16.50	28.90	5.327	0.978	599	n.a	n.a	n.a
PR120106_3S	2012.1.6	113.751	22.454	115.700	n.a	0.600	133.200	0.000	7.90	13.20	26.70	5.606	0.992	579	n.a	n.a	n.a
PR120107_6S	2012.1.7	113.571	22.874	206.100	n.a	0.800	83.300	0.000	7.30	16.10	14.60	5.760	0.985	767	n.a	n.a	n.a
PR120107_7S	2012.1.7	113.160	23.055	827.300	n.a	1.400	200.600	26.600	7.20	15.00	0.40	4.751	0.970	453	n.a	n.a	n.a
PR120109_10S	2012.1.9	112.963	23.090	166.100	n.a	0.400	127.000	0.000	7.70	13.60	0.10	5.340	0.977	625	3.916	188	n.a
PR120109_12S	2012.1.9	112.825	23.161	58.600	n.a	0.800	131.200	1.400	7.50	14.40	0.10	5.041	0.977	527	3.721	176	n.a
PR120109_13S	2012.1.9	112.807	23.219	221.400	n.a	4.600	116.400	0.500	7.50	13.40	0.10	n.a	n.a	n.a	4.259	218	n.a
PR120112_21S	2012.1.12	113.173	23.701	5.900	n.a	5.800	120.600	0.000	7.80	12.50	0.10	4.344	0.956	380	2.068	71	n.a
PR110615AS	2011.6.15	113.704	21.952	n.a	n.a	n.a	n.a	n.a	n.a	n.a	n.a	n.a	n.a	n.a	n.a	n.a	n.a
PR110617BS	2011.6.17	113.751	22.454	n.a	n.a	n.a	n.a	n.a	n.a	n.a	n.a	n.a	n.a	n.a	n.a	n.a	n.a
PR110619CS	2011.6.19	113.571	22.877	n.a	n.a	n.a	n.a	n.a	n.a	n.a	n.a	4.120	0.891	441	n.a	n.a	n.a
scs_0S	2012.10	113.708	21.932	n.a	n.a	n.a	n.a	n.a	n.a	n.a	n.a	5.221	0.980	546	n.a	n.a	n.a
scs_1S	2012.10	113.700	21.500	n.a	n.a	n.a	n.a	n.a	n.a	n.a	n.a	5.270	0.977	626	3.419	143	n.a
scs_2S	2012.10	113.800	21.000	n.a	n.a	n.a	n.a	n.a	n.a	n.a	n.a	5.400	0.985	563	4.197	259	n.a
scs_3S	2012.10	113.900	21.000	n.a	n.a	n.a	n.a	n.a	n.a	n.a	n.a	5.374	0.981	663	4.188	230	n.a
scs_4S	2012.10	114.600	20.000	n.a	n.a	n.a	n.a	n.a	n.a	n.a	n.a	4.400	0.961	379	4.641	278	n.a
scs_5S	2012.10	114.896	19.500	n.a	n.a	n.a	n.a	n.a	n.a	n.a	n.a	4.611	0.960	388	n.a	n.a	n.a
scs_52S	2012.10	112.164	21.047	n.a	n.a	n.a	n.a	n.a	n.a	n.a	n.a	5.532	0.986	613	3.017	156	n.a
scs_57S	2012.10	112.698	20.377	n.a	n.a	n.a	n.a	n.a	n.a	n.a	n.a	6.148	0.994	862	3.892	209	n.a

[a] n.a - not available

[b] Sample names in red represent data from one of our previous studies.

[c] Environmental factors with yellow background represent data from WOA13 (https://odv.awi.de/en/data/ocean/world_ocean_atlas_2013/).

TABLE S2 Designated AOA OTUs based on the *amoA* gene were mapped to a specific evolutionary tree (Alves et al., 2018) to determine their phylogenetic clades.

Major lineages	Subgroup	OTU
NP (Nitrosopumilales)	NP-γ-2.2	OTU13
		OTU83
	NP-γ-2.1	OTU7
		OTU31
		OTU84
		OTU117
		OTU118
		OTU119
	NP-γ-1	OTU15
		OTU32
		OTU55
		OTU89
	NP-α-2.2.4	OTU5
		OTU92
		OTU100
		OTU102
	NP-α-2.2.3	OTU9
		OTU49
		OTU54
	NP-α-2.1	OTU37
		OTU38
		OTU58
		OTU74
		OTU78
		OTU106
		OTU107
	NP-α-1	OTU2
		OTU11
		OTU21
		OTU25
		OTU42
		OTU57
		OTU85
		OTU86
		OTU87
		OTU88
		OTU90
		OTU91
		OTU103
		OTU116

NP-β	OTU34
	OTU72
NP-δ	OTU12
	OTU26
	OTU30
	OTU40
	OTU47
	OTU56
	OTU59
	OTU77
	OTU79
	OTU80
	OTU101
	OTU105
	OTU108
	OTU111
	OTU112
	OTU113
NP-θ	OTU29
	OTU60
	OTU73
	OTU75
	OTU76
	OTU81
	OTU104
	OTU114
	OTU115
NP-η	OTU8
	OTU17
	OTU20
	OTU24
	OTU28
	OTU39
	OTU41
	OTU45
	OTU46
	OTU52
	OTU61
	OTU66
	OTU71
	OTU95
	OTU98
	OTU99
NP-ε	OTU1
	OTU14
	OTU82

		OTU93
		OTU109
		OTU110
		OTU120
		OTU121
	NP-ζ	OTU64
NT (Ca.Nitrosotaleales)	NT-α	OTU4
		OTU69
NS (Nitrososphaerales)	NS-α	OTU19
		OTU23
		OTU35
		OTU44
	NS-γ	OTU33
		OTU36
		OTU67
	NS-β	OTU43
		OTU50
		OTU51
		OTU68
		OTU70
	NS-δ	OTU6
		OTU10
		OTU16
		OTU18
		OTU27
		OTU48
		OTU53
		OTU62
		OTU63
		OTU94
		OTU96
		OTU97
	NS-ε	OTU65
	NS-IS-1	OTU3
		OTU22

TABLE S3 The analysis of similarities (ANOSIM) for total archaeal community composition in OTU level among groups.

	High-salinity-water	Low-salinity-water	High-salinity-sediment	Low-salinity-sediment
High-salinity-water				
Low-salinity-water	R=0.922<br>P=0.001			
High-salinity-sediment	R=0.834<br>P=0.001	R=0.943<br>P=0.001		
Low-salinity-sediment	R=0.963<br>P=0.001	R=0.073<br>P=0.201	R=0.947<br>P=0.001	

TABLE S4 The analysis of similarities (ANOSIM) for AOA composition in OTU level among groups.

	High-salinity-water	Low-salinity-water	High-salinity-sediment	Low-salinity-sediment
High-salinity-water				
Low-salinity-water	R=0.279<br>P=0.021			
High-salinity-sediment	R=0.926<br>P=0.001	R=0.997<br>P=0.001		
Low-salinity-sediment	R=0.841<br>P=0.001	R=0.633<br>P=0.013	R=1.000<br>P=0.006	

TABLE S5 The analysis of similarities (ANOSIM) for AOA composition in OTU level among groups.

Network properties		water			sediment		
		all	high-salinity	low-salinity	all	high-salinity	low-salinity
Empirical network	Nodes	671	396/227	420	818	514/424	359
	Links	2574	1212/611	2933	3113	1773/2286	1809
	Positive	1547	934/415	1265	1818	1191/1508	953
		**(60.10%)**	**(77.06%)/(67.92%)**	**(43.13%)**	**(58.40%)**	**(67.17%)/(65.97%)**	**(52.68%)**
	Negative	1027	278/196	1668	1295	582/778	856
		(39.90%)	***(22.94%)/(32.08%)***	***(56.87%)***	***(41.60%)***	***(32.83%)/(34.03%)***	***(47.32%)***
	Diameter	22	17	13	24	18	13
	Density	0.011	0.015/0.024	0.033	0.009	0.013/0.025	0.028
	avgCC	0.285	0.217/0.232	0.338	0.299	0.271/0.276	0.324
	avgPL	6.065	7.236/6.060	4.665	9.135	6.805/5.207	4.778
	Modularity	0.787	0.774/0.797	0.726	0.792	0.782/0.739	0.708
	R^2 of power-law	0.696	0.647/0.500	0.433	0.720	0.784/0.740	0.501
Random network	$avgCC_r$	0.027	0.023/0.036	0.075	0.020	0.035/0.055	0.054
		(0.003)	(0.004)/(0.007)	(0.005)	(0.002)	(0.004)/(0.005)	(0.005)
	$avgPL_r$	3.417	3.486/3.365	2.756	3.510	3.369/2.934	2.898
		(0.021)	(0.022)/(0.034)	(0.017)	(0.016)	(0.025)/(0.020)	(0.020)
	Modularity	0.317	0.370/0.394	0.205	0.323	0.339/0.246	0.261
		(0.004)	(0.005)/(0.007)	(0.004)	(0.004)	(0.004)/(0.004)	(0.005)

[a] Number of OTUs with the correlation $|r| > 0.6$ and statistical significance ($P < 0.05$).

[b] In the empirical network, the numbers in parentheses represent the proportion of positive correlations in the network, and the italic numbers represent the proportion of negative correlations.

[c] In random network, the numbers in parentheses represent the standard deviation.

[d] avgCC, average clustering coefficient; avgPL, average path length; r, random network.

[e] In the high-salinity subnetworks, the numbers in front of the slashes represent the topological eigenvalues calculated using all high-salinity samples; The numbers behind the slashes represent the topological eigenvalues obtained by randomly sampling the same number of samples from the high-salinity groups as from the low-salinity groups.

TABLE S6 Number of degrees of different archaeal groups in eight major modules in planktonic archaeal network from the PR to the NSCS.

	Module 1 (10.29%)	Module 2 (9.84%)	Module 3 (8.35%)	Module 4 (7.15%)	Module 5 (6.26%)	Module 6 (5.51%)	Module 7 (5.37%)	Module 8 (5.22%)
Marine Group II	0	0	**185**	0	0	0	39	**110**
Candidatus Nitrosopumilus	0	0	0	20	0	0	4	**22**
Others	263	152	6	73	59	113	40	11
Woesearchaeales	**335**	110	16	56	16	**104**	**145**	8
Marine Group III	0	0	**58**	0	0	0	0	6
Bathyarchaeia	**327**	**227**	0	**258**	9	**61**	**179**	5
Nitrosopumilaceae	60	0	0	20	0	0	19	0
CG1-02-57-44	26	8	0	0	0	0	19	0
Methanosaeta	32	**123**	0	**80**	**16**	17	17	0
Methanoregula	36	43	0	2	13	29	0	0
Nitrosotaleaceae	45	0	0	0	0	17	0	0
Candidatus Methanomethylicus	23	0	0	0	0	9	0	0
Candidatus Methanoperedens	30	0	0	0	0	7	0	0
Methanolinea	0	32	0	21	11	4	0	0
Methanobacterium	0	28	1	2	**28**	0	0	0
Marine Benthic Group D	24	10	0	20	9	0	0	0
Marine Benthic Group A	15	16	0	0	5	0	0	0
Lokiarchaeia	15	40	0	20	0	0	0	0
ANME-2a-2b	0	22	0	11	0	0	0	0
Candidatus Caldiarchaeum	21	11	0	0	0	0	0	0

TABLE S7 Number of degrees of different archaeal groups in eight major modules in benthic archaeal network from the PR to the NSCS.

	Module 1 (10.52%)	Module 2 (9.54%)	Module 3 (9.05%)	Module 4 (7.82%)	Module 5 (7.21%)	Module 6 (6.85%)	Module 7 (5.75%)	Module 8 (5.62%)
Bathyarchaeia	**176**	**220**	**123**	**374**	**152**	**137**	**104**	24
Woesearchaeales	**80**	**103**	**124**	**149**	**40**	**213**	**91**	**50**
Methanosaeta	0	**116**	**101**	0	0	**102**	**62**	0
Nitrosopumilaceae	16	0	0	0	0	9	0	**153**
Methanoregula	0	43	20	0	0	57	18	0
Candidatus Nitrosopumilus	51	0	0	**24**	24	0	0	25
Marine Benthic Group D	5	0	12	15	**56**	20	15	0
Marine Benthic Group A	0	8	0	20	0	31	1	**63**
Lokiarchaeia	3	0	6	20	30	17	20	4
Candidatus Methanoperedens	0	31	0	0	0	51	0	0
Nitrososphaeraceae	0	47	0	0	0	34	0	0
Methanosarcina	0	17	11	0	0	9	20	0
Hydrothermarchaeales	**55**	0	0	1	0	0	0	0
Methanobacterium	0	13	39	0	0	0	2	0
Methanolinea	0	29	6	0	0	3	15	0
Candidatus Methanomethylicus	0	41	7	0	0	0	0	0
Hadarchaeales	0	0	23	0	0	0	0	8
ANME-1b	0	0	12	19	0	0	0	0
Methanosphaerula	0	0	0	0	0	26	0	0
Others	40	168	139	138	82	161	117	25

TABLE S8 Mean habitat niche breadth for all taxa among the high-salinity-water, low-salinity-water, high-salinity-sediment, and low-salinity-sediment groups.

Community	high-salinity-water	low-salinity-water	high-salinity-sediment	low-salinity-sediment
Bcom	3.0536	2.3344	2.8388	2.565
